# Modified MXene Aerogel With Broadband Microwave Absorption Inspired by Melanophila Acuminata Beetle

**DOI:** 10.1002/advs.76496

**Published:** 2026-07-13

**Authors:** Zhiwei Liu, Dingyu Xu, Zhaobo Liu, Haitian Song, Qianqian Zhang, Junxiao Zhou, Xiewen Wen

**Affiliations:** ^1^ Department of Industrial and Systems Engineering The Hong Kong Polytechnic University Kowloon Hong Kong SAR China; ^2^ The Hong Kong Polytechnic University Shenzhen Research Institute Shenzhen China; ^3^ The Hong Kong Polytechnic University Wuxi Technology and Innovation Research Institute Wuxi China; ^4^ School of Materials Science and Engineering Beihang University Beijing China; ^5^ Fujian Academy of Forestry Fuzhou China; ^6^ National Laboratory of Solid State Microstructures Key Laboratory of Intelligent Optical Sensing and Manipulations Jiangsu Key Laboratory of Artificial Functional Materials Collaborative Innovation Center of Advanced Microstructures College of Engineering and Applied Sciences Nanjing University Nanjing China; ^7^ University Research Facility in 3D Printing The Hong Kong Polytechnic University Kowloon Hong Kong SAR China

**Keywords:** aerogel, MXenes, biomimetic materials, broadband absorption, microwave absorber

## Abstract

The rapid development of wireless technologies has led to significant electromagnetic interference (EMI), which can damage sensitive electronic equipment and cause potential biological safety issues. Conventional electromagnetic wave (EMW) absorbing materials are often hindered by a series of complicated limitations, including high density, poor impedance matching, and narrow absorption bandwidths, which limit their use in advanced applications. To overcome these challenges, this work introduces a novel biomimetic strategy inspired by the infrared‐sensing sensilla of the Melanophila acuminata beetle. We have designed a multi‐level aerogel, featuring a three‐dimensional carbon framework decorated with synergistic CeO_2_ nanoparticles and Ti_3_C_2_T_x_ MXene nanosheets. This design creates abundant heterogeneous interfaces and absorption paths and utilizes complementary loss mechanisms to achieve outstanding performance, validated by theoretical analysis. The resulting aerogel achieves a record‐breaking effective absorption bandwidth of 9.43 GHz with only 3.74 wt.% filler loading, establishing a new benchmark for lightweight, broadband electromagnetic absorption. This work presents a promising design paradigm for developing advanced EMW absorbing materials with significant potential for next‐generation stealth technologies and EMI shielding.

## Graphical abstract

This work introduces a novel biomimetic strategy inspired by the infrared‐sensing sensilla of the Melanophila acuminata beetle. We have designed a multi‐level aerogel, featuring a three‐dimensional carbon framework decorated with synergistic CeO_2_ nanoparticles and Ti_3_C_2_T_x_ MXene nanosheets. The resulting aerogel achieves a record‐breaking effective absorption bandwidth of 9.43 GHz with only 3.74 wt.% filler loading.

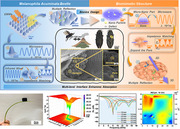

## 1 Introduction

The increasing use of wireless communication technologies, portable electronics, and complex radar systems in the gigahertz (GHz) spectrum has led to a significant increase in electromagnetic radiation [1, 2]. This pervasive electromagnetic interference (EMI) not only compromises the stability and performance of sensitive electronic apparatus but also raises growing concerns regarding potential impacts on biological systems [3]. Consequently, the development of high‐performance electromagnetic wave (EMW) absorbing materials characterized by attributes such as low density, broad effective absorption bandwidth (EAB), and strong absorption capabilities has become a critical scientific and technological imperative [4, 5]. While conventional EMW absorbing materials, including ferrites and metallic powders, have been investigated, they often have limitations such as narrow absorption bandwidths, high densities, and inadequate impedance matching, thus curtailing their efficacy in advanced applications [6].

Nature, through millennia of evolutionary optimization, presents a source of inspiration for the design of functional materials with intricate, hierarchically organized structures [7]. A notable example is the *Melanophila acuminata* beetle, renowned for its extraordinary infrared (IR) sensory organs capable of detecting distant forest fires. These sensilla exhibit a complex, multi‐layered architecture incorporating dome‐like cuticular lenses, internal micro‐cavities, and nanoporous wax filaments, which collectively enable efficient, broadband IR radiation trapping and absorption [8]. Emulating such natural hierarchical paradigms, which leverage principles of multiple scattering, gradient refractive indices, and maximized interfacial interactions to enhance wave‐matter coupling, offers a promising strategy for engineering EMW absorbing materials with significantly amplified performance.

The intrinsic electromagnetic properties of constituent materials are critical in dictating the overall absorption efficacy. Cerium dioxide (CeO_2_), a rare‐earth oxide possessing a distinctive electronic structure and a high concentration of oxygen vacancies, has garnered substantial interest as a dielectric loss medium [9]. These oxygen vacancies can serve as polarization centers, promoting dipole polarization and enhancing dielectric loss mechanisms under incident EMWs, particularly when engineered at the nanoscale [10]. Concurrently, MXenes, an emergent class of two‐dimensional (2D) transition metal carbides and/or nitrides (e.g., Ti_3_C_2_T_x_, where Tx denotes surface terminations such as ‐O, ‐OH, ‐F), have advanced the field of EMW absorption [11, 12]. Their unique combination of metallic conductivity, abundant functional groups facilitating interfacial polarization, lamellar structure enabling multiple internal reflections, and excellent mechanical properties makes them exceptionally promising candidates for EMW attenuation [13, 14]. The three‐dimensional aerogel structure can effectively disperse the nanomaterials, thereby demonstrating its significant potential for electromagnetic wave absorption [15, 16, 17, 18]. The synergistic integration of CeO_2_, with its pronounced dielectric loss, and MXene, with its potent conductive and interfacial polarization losses, is anticipated to yield hybrid materials with complementary absorption mechanisms, thereby achieving superior EMW attenuation across a broader frequency spectrum [19].

Herein, we report the rational design and synthesis of a novel, bio‐inspired hierarchical aerogel, CeO_2_/MXene@CF_m_. Its structural design draws inspiration from the IR sensilla of the *Melanophila acuminata* beetle. The porous structure of aerogels results in a large portion being occupied by air, which facilitates impedance matching with air and minimizes reflection at the incident interface. At the same time, the high porosity can extend the propagation path of the incident EMWs, and repeated collisions with the absorbing components can increase the absorption capacity [20, 21, 22]. Moreover, the ability to attenuate EMWs is jointly contributed by the ion defects from CeO_2_, the functional sites in MXene, and the carbon defects obtained through the sacrificial template method [23, 24]. The in‐situ self‐assembly of 0D nanoparticles, 2D nanosheets, and 3D skeleton structure provides an extremely rich heterogeneous interface to further expand the attenuation effect of EMWs. It is worth noting that the abundant attenuation levels and different‐sized pores are expected to significantly expand the EAB. Notably, the lightweight (density: 0.018∼0.117 g cm^−3^) aerogel achieves a remarkable EAB of 9.43 GHz (2‐18 GHz, only 3.74wt.% of epoxy resin) at a thickness of 3.9 mm, exceeding the performance of single absorbing components reported to date. This ultra‐wideband absorption, coupled with its low density, positions CeO_2_/MXene@CF_m_ as a highly promising material solution for next‐generation EMI shielding and stealth technologies, addressing the critical need for high‐performance absorbers operating over extensive frequency ranges.

## 2 Results and Discussion

**FIGURE 1:**
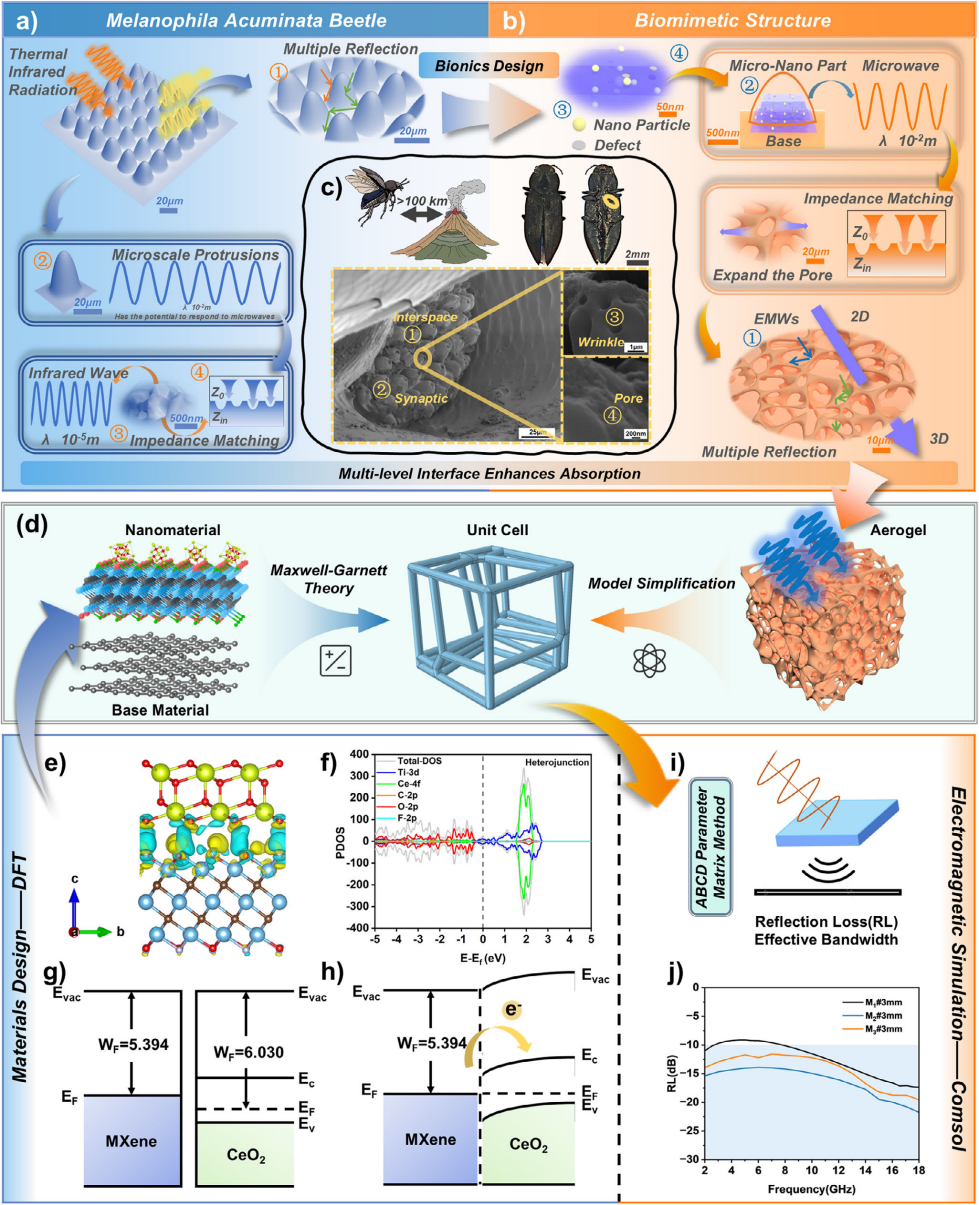
The design and simulation of biomimetic aerogel inspired by the *Melanophila acuminata* beetle. (a) The ultra‐long‐range infrared detection mechanism of *Melanophila acuminata* beetle; (b) Structure‐function‐mechanism mapping of the biomimetic aerogel design; (c) The schematic diagram of the ultra‐long‐range infrared detection, digital photos and SEM images of *Melanophila acuminata* beetle; (d) The schematic diagram of design and simulation of biomimetic aerogel; (e) The schematic diagram of the differential charge density, (f) DOS and (g,h)energy levels of the CeO_2_ and MXene heterojunction; (i) The COMSOL simulation method and (j) result of EMW absorption by biomimetic aerogel.

The remarkable ability of the *Melanophila acuminata* beetle to detect distant forest fires, often from over a hundred kilometers away, is due to specialized IR sensilla—sophisticated natural photodetectors housed within thoracic pit organs, as shown in Figure 1c. We used scanning electron microscopy (SEM) to study the ultrastructure of these IR sensilla, revealing a complex, multi‐scale hierarchical architecture. These sensilla detect thermal infrared radiation rather than microwaves, but their hierarchical architecture provides a valuable biological prototype for wave‐management design. As shown in Figure 1a, when combined with the SEM image in Figure 1c, it can be observed that the IR sensilla have particularly densely arranged “synaptic‐like” structures (Feature ②), and there are gaps between the ordered synaptic structures (Feature ①). The pores between the synaptic structures provide paths for multiple reflections and scattering of thermal infrared radiation, allowing the sensilla to have more contact with the radiation. The micrometer‐sized synaptic structures mainly resonate in the microwave band and are insensitive to infrared and far‐infrared bands. What truly receives (far) infrared signals is the nano‐scale wrinkles on the surface of the synaptic structures (Feature ③). The nano‐scale structure can resonate with (far) infrared waves, receive signals and convert them into physiological information. In addition, nano‐scale holes are scattered between the nano‐scale wrinkles (Feature ④), which makes the impedance characteristic of the sensilla structure closer to air, promoting the perception of EMW signals. In the natural sensillum, these multiscale structures are associated with infrared radiation trapping, enlarged wave‐matter interaction area, gradual impedance transition between air and the sensing organ, and efficient conversion of thermal radiation into biological signals. This natural wonder directly inspired us to develop advanced EMW absorption metamaterials.

It should be emphasized that the present biomimetic strategy is based on functional translation rather than direct geometric duplication. The infrared‐sensing sensilla of Melanophila acuminata naturally operate in the infrared/far‐infrared regime, whereas the present aerogel is designed for microwave attenuation in the 2–18 GHz band. Therefore, the biological structure is used as a wave‐management prototype that provides several transferable design principles, including hierarchical impedance regulation, enhanced radiation trapping, prolonged propagation pathways, and interfacial energy conversion.

Figure 1b illustrates the logical principle of our design of EMW absorption metamaterials through a biomimetic strategy. Following this principle, the interspaces and cavities in the sensilla are translated into a 3D porous carbon framework, which lowers the effective permittivity, improves impedance matching with free space, and allows incident microwaves to enter the absorber (Feature ①). The synaptic‐like protrusions are translated into rough interconnected carbon struts decorated with CeO_2_/MXene components, increasing the wave‐material interaction area and elongating the propagation path (Feature ②). Meanwhile, the nanoscale wrinkles and pores of the biological structure inspire the construction of abundant CeO_2_/MXene/carbon heterointerfaces, oxygen vacancies, and MXene surface terminations, which serve as polarization and loss centers through Maxwell‐Wagner interfacial polarization, defect dipole relaxation, and conductive loss [25], (Feature ③④). The overall hierarchical architecture further contributes to multiscale impedance regulation and broadband attenuation. Thus, the beetle‐inspired design establishes a clear biological feature‐physical function‐microwave attenuation relationship for broadband EMW absorption. We preserved the beetle's superior strategy of hierarchical impedance regulation and interfacial energy dissipation but rationally adjusted the characteristic dimensions to align with the microwave physics of the 2–18 GHz band.

To assess the electromagnetic (EM) response of this bio‐inspired structure and to verify its design principles before experimental fabrication, we conducted comprehensive EM simulations (Figure 1d). Firstly, we simplify the aerogel into a framework structure of an irregular Voronoi polyhedron with a micrometer size, and then break it down into a single unit cell. The specific geometric parameters of this unit cell, along with the precise frequency‐dependent complex permittivity (*ε_r_​ = ε′−jε′′*) and permeability (*µ_r_​ = µ′−jµ′′*) values of the constituent materials derived using the Maxwell‐Garnett Theory, are comprehensively detailed in the Supplementary Information [26]. The reason for choosing this structure is not only its similar shape, but also its irregular structure is beneficial for EMW absorption. Based on this, we add materials to the structure, using the framework structure as the base, and perform Density Functional Theory (DFT) calculations on the selected micro‐nano materials to confirm their excellent characteristics. Then, we conduct COMSOL electromagnetic simulation on the post‐materialized single unit cell.

The material components that form the multi‐level structure mainly determine the EMWs absorption capacity. To realize this biomimetic concept, we strategically selected CeO_2_ and Ti_3_C_2_T_x_ MXene as primary nanomaterial building blocks. Their choice was driven by their complementary dielectric and conductive properties, which are crucial for EMW absorption. Moreover, combining these two nanomaterials inherently introduces multi‐level characteristics at the nanoscale, crucially providing abundant heterogeneous interfaces for EMW dissipation. This dual‐component approach is also supported by existing literature, which suggests a potential synergistic effect between CeO_2_ and MXene in EMW absorption [19]. A key aspect of our modeling involved representing the MXene phase as Ti_3_C_2_OF. This specific stoichiometry wasn't arbitrarily chosen; it was determined by the quantitative elemental analysis of the MXene material synthesized in our experiments, thereby ensuring our theoretical model directly correlated with the actual experimental system (Table S1). Furthermore, the atomic configuration of the CeO_2_/Ti_3_C_2_OF heterojunction model used for these calculations was established. We systematically constructed and evaluated various possible interfacial arrangements, and through comparative binding energy calculations, identified the most stable configuration. (Figures S1 and S2 and Table S2).

The differential charge density (DCD) analysis of the CeO_2_/Ti_3_C_2_OF interface (Figure 1e) revealed a significant interfacial charge redistribution, with electron accumulation predominantly on the CeO_2_ side and a corresponding electron depletion on the Ti_3_C_2_OF MXene side. This substantial charge transfer signifies strong electronic coupling between the constituent materials and leads to the formation of a built‐in electric field and prominent interfacial dipoles at the heterointerface. Such pronounced charge polarization is expected to enhance dielectric loss, primarily through interfacial polarization (Maxwell‐Wagner effect) and subsequent relaxation mechanisms under an alternating electromagnetic field, which are crucial for attenuating EMWs.

Analysis of the electronic Density of States (DOS) provided further insights into the electronic structure of the heterojunction (Figure 1f). A general enhancement in the projected density of states (PDOS) was observed for the CeO_2_/Ti_3_C_2_OF heterojunction compared to its individual constituents, suggesting an increased number of available electronic states arising from the strong interfacial coupling (Figure S3). Notably, the states at and near the Fermi level were found to be predominantly contributed by the Ti 3d orbitals of the Ti_3_C_2_OF MXene component. This characteristic indicates that the heterostructure likely retains or possesses good intrinsic electronic conductivity, channeled primarily through the MXene layers, which is essential for promoting conductive loss mechanisms when interacting with EMWs, as mobile electrons in these Ti 3d states can efficiently dissipate energy via Joule heating. Further examination of the PDOS revealed significant orbital hybridization at the interface, evidenced by the considerable overlap of Ce 4f, Ti 3d, O 2p, and F 2p electronic states, particularly in the energy range above the Fermi level. These newly formed unoccupied hybridized states provide accessible energy levels for electrons excited by incident EMWs. Such electronic transitions from occupied states below the Fermi level to these unoccupied hybridized states constitute an important channel for energy dissipation and enhanced dielectric loss. These theoretical results collectively indicate that CeO_2_/MXene heterojunctions possess intrinsic electronic and interfacial characteristics—namely strong interfacial polarization, optimized charge transport channels, and an electronic structure tailored for enhanced energy conversion—that are highly beneficial for superior EMW absorption.

Furthermore, calculations of the work functions (Φ) for the individual components were important for understanding charge dynamics at the heterojunction. As shown in Figures S4 and S5 the work function for the lower surface of CeO_2_ was determined to be Φ_CeO2​​_ = 6.030 eV, while for the upper surface of Ti_3_C_2_OF, it was Φ_Ti3​C2​OF_​ = 5.394 eV. Given that Φ_CeO2_​​>Φ_Ti3​C2​OF_​, upon formation of the heterojunction, electrons are driven to flow from Ti_3_C_2_OF to CeO_2_ to achieve Fermi level alignment (Figure 1g,h). This directional electron transfer results in an accumulation of negative charge on the CeO_2_ side and positive charge (electron depletion) on the Ti_3_C_2_OF side of the interface. Consequently, a strong built‐in electric field is established, pointing from Ti_3_C_2_OF toward CeO_2_. This internal field is highly beneficial for EMW absorption as it can effectively promote the separation of field‐induced charge carriers, reducing their recombination and enhancing conductive loss. Moreover, the pronounced charge separation at the interface significantly strengthens the aforementioned interfacial polarization, further contributing to dielectric losses.

Our simulations systematically calculated the scattering parameters (S_11_ and S_21_) for the unit cell across the user‐defined input EM field frequency range (2–18 GHz). In the sub‐wavelength limit, where the structural inhomogeneities are significantly smaller than the operating wavelength, a complex medium can be accurately described by its effective constitutive parameters (*ɛ* and *µ*) [27]. Due to this sub‐wavelength nature, complex diffraction and edge scattering effects are physically suppressed. Therefore, employing a periodic Voronoi unit cell to represent the equivalent refractive index and macroscopic dielectric loss is a rigorous and standard approach in metamaterial research. To extrapolate the unit cell's performance to a macroscopic sample with a defined thickness (3 mm in the Z‐direction), we applied transmission line theory and the ABCD matrix method (Figure 1i). The specific plan is provided in the Supplementary Information. Finally, the reflection loss (RL), which quantifies the amount of incident EM wave power reflected from the material's surface.

These computational outputs (Figure 1j) predicted robust microwave absorption within the 2–18 GHz range, with over 90% EM wave absorption (RL < −10 dB) across nearly the entire band. This forecasts a significant advantage for our design in terms of effective absorption bandwidth. While COMSOL simulation applies periodic repetition in the X and Y directions, and the ABCD matrix method stacks units simply in the Z‐direction, representing a basic repetition of the unit cell, in actual samples, we can enhance EM wave absorption by increasing disorder in XYZ space to complicate EM wave propagation paths. Concurrently, improved connectivity within the skeletal structure can further boost its conductivity [28].

**FIGURE 2:**
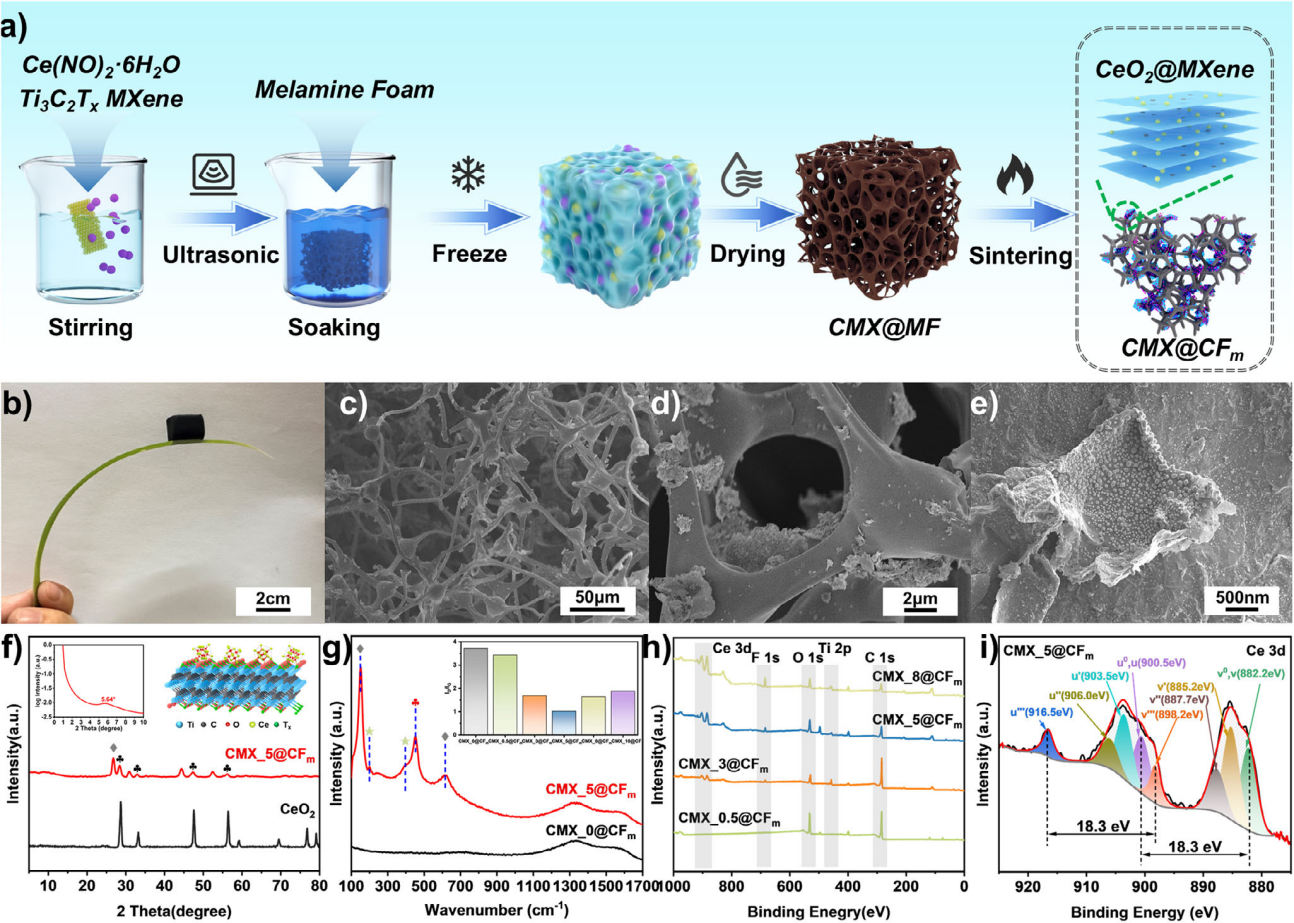
The preparation and microscopic properties of biomimetic electromagnetic aerogel. (a) The preparation flowchart of biomimetic electromagnetic aerogel; (b) The digital photograph of lightweight biomimetic electromagnetic aerogel (on a small grass blade); (c‐e) The SEM images, (f) XRD spectra and SAXS spectrum, (g) Raman spectra, (h) XPS spectra and (i) Ce 3d spectra of biomimetic electromagnetic aerogel.

Overall, our work draws inspiration from the complex, multi‐level IR sensilla of the *Melanophila acuminata* beetle. Central to this design is a 3D carbon framework aerogel adorned with CeO_2_/MXene nanosheets. The core principle exploits the synergy among dielectric CeO_2_, conductive MXene, and the porous carbon scaffold, all precisely orchestrated within a multi‐scale architecture. The rational design of such heterostructures, via interfacial self‐assembly, enables the creation of 3D arrays, further amplifying the interfacial polarization and scattering effects, ultimately leading to superior EM wave absorption performance across a wide frequency range. Importantly, the dual‐component nanomaterials themselves introduce multi‐level characteristics even at the nanoscale, providing abundant heterogeneous interfaces critical for EMW dissipation, a synergy further corroborated by existing literature [29]. The specific experimental strategy to realize this bio‐inspired aerogel, spanning precursor selection to final aerogel formation, is systematically outlined in the synthesis flowchart (Figure 2a). We strategically chose melamine foam (MF) as a sacrificial template due to its inherent skeletal structure and pore dimensions closely mimicking our theoretically established Voronoi‐like porous design. Its capacity to effectively adsorb nanomaterial precursors for integrated synthesis was also pivotal. Furthermore, the intrinsic random nature of its skeletal structure is anticipated to further augment EMW absorption capabilities.

Novel CeO_2_/Ti_3_C_2_T_x_ MXene‐decorated carbon framework aerogels (CMX@CF_m_) were successfully synthesized, experimentally realizing a design paradigm previously optimized through DFT calculations and COMSOL simulations for superior EMW absorption. These aerogels, presenting as ultralight materials with densities ranging from 0.018 to 0.117 g/cm^3^ (Table S3), could rest on delicate structures like flower stamens (Figure 2b). The aerogels exhibited controlled volumetric shrinkage (5.60% to 13.07%, Table S3) during their formation.

The morphology and internal architecture of the CMX@CF_m_ aerogels were investigated using SEM (Figures S6 and S7). Optimal loading levels like CMX_5@CF_m_ led to a uniform and substantial coating of CeO_2_/MXene nanosheets, forming micrometer‐scale, sheet‐like agglomerates enveloping the carbon struts (Figure 2c–e). This configuration significantly increased the density of heterojunction interfaces within the aerogel while substantially preserving its 3D porous architecture, thereby providing the hierarchical geometry predicted by COMSOL simulations to enhance impedance matching and facilitate multiple scattering of incident EMWs. Typically, CMX@CF_m_ aerogels synthesized with optimal loadings featured well‐dispersed CeO_2_/MXene nanosheets, creating abundant interfaces with the carbon framework. These interfaces are critical, as DFT calculations indicated that such CeO_2_/MXene heterojunctions would exhibit significant interfacial charge redistribution, forming dipoles conducive to enhanced dielectric loss under an electromagnetic field.

The crystalline phases within the CMX@CF_m_ aerogels, which underpin the intrinsic material properties used in simulations, were identified using x‐ray diffraction (XRD) and small‐angle x‐ray scattering (SAXS). The carbon framework itself (CMX_0@CF_m_) showed disordered graphitic carbon (Figure S8). As shown in Figure 2f, In the CMX_5@CF_m_ aerogel, A set of distinct peaks appearing at 28.4°, 32.9°, 47.4°, and 56.3° confirms the presence of cubic CeO_2_ (JCPDS No. 34–0394), corresponding to the (111), (200), (220), and (311) crystal planes, respectively. Other peaks were attributed to minor TiO_2_. SAXS analysis of CMX_5@CF_m_ (the inset of Figure 2f) revealed a peak at 5.64°, corresponding to the (002) plane of Ti_3_C_2_T_x_ MXene, confirming its incorporation. As shown in Figure S9, Raman spectroscopy revealed key molecular vibrational information within the aerogel. Raman spectroscopy (Figure 2g) of CMX_5@CF_m_ displayed peaks confirming CeO_2_ (452 cm^−^^1^), Ti_3_C_2_T_x_ MXene (around 200 and 390 cm^−^^1^), and titanium oxides (151^1^, 615 cm^−^^1^). The carbonaceous framework was confirmed by D and G bands (1329–1393 cm^−^^1^ and 1533–1580 cm^−^^1^). The inset in Figure 2g indicates that the I_D_/I_G_ value of the aerogel decreased after the loading of CeO_2_/MXene nanosheets, attributable to the introduction of the more ordered MXene carbon structure.

XPS analyzed the surface elemental composition and chemical states (Figure 2h). The CMX@CF_m_ aerogel clearly showed Ti 2p and Ce 3d signals, confirming the surface presence of Ti_3_C_2_T_x_ MXene and CeO_2_. A high‐resolution XPS scan of the Ce 3d region for CMX_5@CF_m_ confirmed the coexistence of Ce^3^^+^ and Ce^4^^+^ states. As observed in Figure 2i, the Ce 3d spectrum typically consists of two main spin‐orbit split components: 3d*^5/2^ and 3d* ^3/2^. The prominent energy separation of approximately 18.3 eV between these two multiplets is characteristic of the spin‐orbit coupling of the Ce 3d core level, a fundamental atomic property that results from the interaction between the orbital angular momentum (l = 2) and spin angular momentum (s = ±1/2) of the core electrons [30]. The coexistence of Ce^3+^ and Ce^4+^ in cerium oxide is one of its unique electronic structural characteristics. Ce^4+^ has a stable 4f^0^ electron configuration, while Ce^3+^ has a 4f^1^ electron configuration [31, 32]. The presence of Ce^3+^ introduces additional electronic energy levels, thereby altering the material's band gap structure and electron transport properties. When EMWs impinge on the material's surface, incident photons can excite these electrons to transition from lower to higher energy levels, leading to polarization loss. The significant presence of Ce^3^^+^ indicates a high concentration of oxygen vacancies within the CeO_2_ lattice. The presence of oxygen vacancies is often accompanied by local charge imbalance and lattice distortions, which introduce defect dipoles within the material [33, 34, 35, 36]. These defect dipoles relax under the influence of an alternating electromagnetic field, leading to dipole relaxation loss, effectively converting EMW energy into heat. In addition, the high‐resolution Ti 2p XPS spectra (Figure S10) exhibit a characteristic spin‐orbit doublet, indicating the presence of a small amount of titanium dioxide (TiO_2_). The minor presence of TiO_2_ in aerogel significantly enhances EMW absorption primarily by augmenting interfacial polarization loss at the newly formed heterogeneous interfaces.

This comprehensive characterization confirms the formation of CeO_2_/MXene‐decorated carbon framework aerogels. This experimentally realized hierarchical architecture, rich in CeO_2_/MXene interfaces and CeO_2_ oxygen vacancies, provides the physical and chemical foundation for the synergistic EMW absorption mechanisms—including interfacial polarization at heterojunctions (Maxwell‐Wagner effects), dipole polarization related to oxygen vacancies, and conductive losses from the MXene/carbon network—that were predicted and elucidated by our prior DFT and COMSOL simulation studies. The definite presence of the porous structure ensures the possibility of multiple reflections and scattering of EMWs.

**FIGURE 3:**
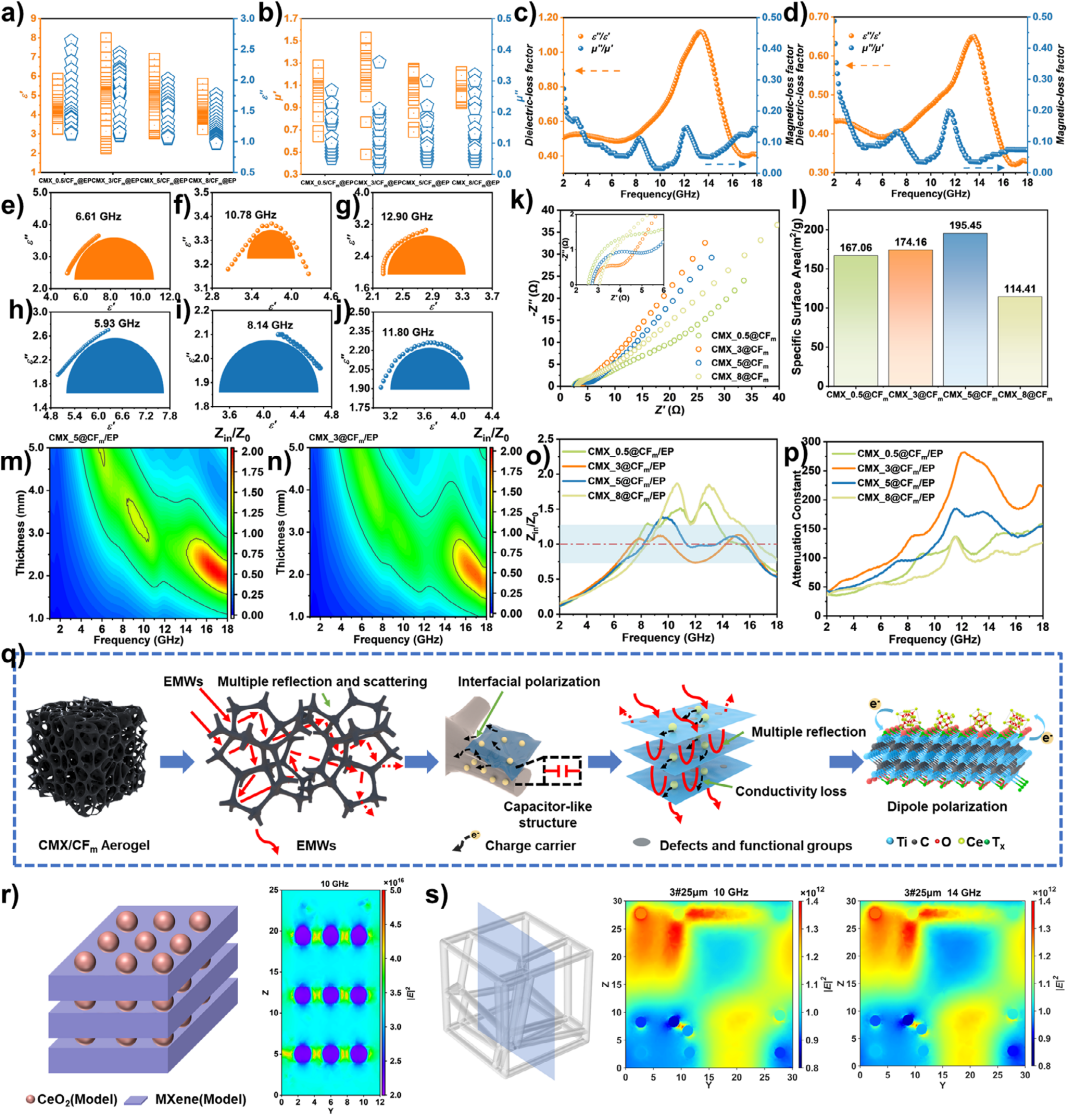
The EMW absorption mechanism of biomimetic electromagnetic aerogel. The relationship between the real part and imaginary part of the relative dielectric constant and the real part and imaginary part of the relative permeability of the biomimetic electromagnetic aerogel: (a,b) comparison of four biominetic electromagnetic aerogels, (c) CMX_3@CF_m,_(d) CMX_5@CF_m_, The Cole‐Cole semicircles of (e‐g) CMX_3@CF_m_ and(h‐j) CMX_5@CF_m_; (k) The impedance spectra and (l) the specific surface area of CMX@CF_m_; Two‐dimensional impedance matching diagram of biomimetic electromagnetic aerogel: (m) CMX_3@CF_m_ and(n) CMX_5@CF_m;_ (o) the impedance matching diagram and (p) the attenuation coefficient diagram of biomimetic electromagnetic aerogel; (q) Schematic diagram of the EMW absorption mechanism of biomimetic electromagnetic aerogel. (r,s) Simulation results of electric field distribution between CeO_2_ and MXene using COMSOL Multiphysics.

The EMW absorption performance of the CMX@CF_m_ aerogels were tested after it was supported by epoxy resin filling (CMX@CF_m_/EP). Wetting tests (Figure S11) showed the feasibility of impregnating the aerogel with epoxy resin and SEM images of CMX@CF_m_/EP (Figure S12) confirmed good impregnation. A detailed analysis of *ε_r_* and *µ_r_* provides fundamental insights into the EMW absorption mechanisms, as shown in Figure S13. For the CMX@CF_m_ aerogels, dielectric loss is a predominant contributor. As observed in Figure 3a, with the enhanced loading of nanomaterials within the aerogel, a 3D ordered superstructure formed. This formation directly led to a significant increase in the overall complex permittivity of the samples, indicating that the 3D ordered superstructure enhanced the material's dielectric response in an electromagnetic field. Magnetic losses are considered less dominant given the non‐magnetic nature of the primary components. In contrast, the complex permeability of the material remained largely unchanged, as shown in Figure 3b, due to the material's inherently low magnetism.

The efficiency of energy dissipation in a material subjected to alternating electromagnetic fields is quantified by its dielectric loss tangent (tanδ_ϵ_) and magnetic loss tangent (tanδ_µ_).These are defined as the ratios of the imaginary to real parts of the *ε_r_* and *µ_r_*, respectively:

(1) $$\tan\delta_{\varepsilon }=\frac{\varepsilon^{\prime \prime }}{\varepsilon^{\prime }}$$

(2) $$\tan\delta_{\mu }=\frac{\mu^{\prime \prime }}{\mu^{\prime }}$$

In frequency‐dependent plots of these loss tangents, overlapping peaks between tanδ_ϵ_ and tanδ_µ_ are a critical indicator. Such overlap signifies strong magneto‐dielectric coupling loss, which typically correlates with robust EMW absorption at the corresponding frequencies [37]. As illustrated in Figure 3 c,d, both CMX_3@CF_m_/EP and CMX_5@CF_m_/EP exhibit a prominent peak in their tanδ_ϵ_ within the 2–18 GHz range, specifically located around 12–14 GHz. Concurrently, their tanδ_µ_ displays two distinct peaks, positioned at approximately 8 and 12 GHz, respectively. For CMX_3@CF_m_/EP, a critical observation is the near‐perfect overlap between the tanδ_ϵ_ peak (12–14 GHz) and one of the tanδ_µ_ peaks (around 12 GHz). This significant overlap strongly indicates the presence of robust magneto‐dielectric coupling, which is crucial for efficient EMW absorption. Furthermore, the observed shifts in the peak positions demonstrate that the effective electromagnetic absorption range can be precisely tuned by adjusting the material's composition.

Dielectric relaxation, a fundamental process by which materials dissipate electromagnetic energy, is often characterized by the relationship between the ε′ and ε′′. This relationship, for a single relaxation process, forms a semicircle in a Cole‐Cole plot, known as a Debye semicircle [38]. For the CMX_3@CF_m_/EP (Figure 3e–g) and CMX_5@CF_m_/EP (Figure 3h–j) samples, analysis of their complex permittivity, specifically plotting the ε′′ against ε′, revealed four distinct Debye semicircles. Each semicircle directly corresponds to a specific dielectric relaxation process occurring within the material, contributing to the overall EMW absorption. As shown in Figure S14, the presence of multiple semicircles indicates a complex dielectric response, deviating from an ideal single Debye relaxation and suggesting the involvement of several energy dissipation mechanisms. Similarly, electrochemical Impedance Spectroscopy (EIS) provides profound insights into the underlying EMW absorption mechanisms. Typical Nyquist plots (Figure 3k) distinctly reveal semicircles, indicative of various relaxation processes. The phase angle or reactance arc in EIS reflects the charge storage capacity of the hetero‐interface. This interface characteristic is the prerequisite for generating Maxwell‐Wagner polarization in the GHz frequency band. At higher frequencies, the observed smaller semicircle signifies a remarkably low charge transfer resistance, consistent with the highly conductive MXene and carbon networks (as presented in Table S4), thereby facilitating significant conduction loss. Conversely, at lower frequencies, a more vertical line indicates enhanced ion diffusion and/or charge accumulation within the porous network [39].

Beyond electronic contributions, in this work, the numerous interfaces generated by aerogel's porous structure and multilevel structure are paramount for efficient EM wave absorption [40, 41]. Firstly, characterized by extremely low density, high porosity, and a vast internal surface area, the interconnected porous network significantly enhances EM wave absorption. It achieves this by elongating the propagation path of incident EMWs, thereby increasing the probability of wave‐material interaction and subsequent attenuation. Equally important, this high specific surface area also provides ample sites for the deposition of active absorbing components (CeO_2_@MXene), maximizing the density of heterointerfaces. This multi‐level architecture integrates nanoscale features with microscale structures, creating a plethora of heterointerfaces, including numerous heterojunction interfaces and carbon material interfaces (Figure 3l) and fostering a favorable impedance distribution. The multiple reflections and scattering of EMWs within the aerogel increase the chances of contact with the absorbing components. Subsequently, a large amount of interface polarization mixed with dielectric polarization absorbs the EMWs to the greatest extent. This collective effect initiates a cascade of EMW loss mechanisms, underscoring our initial design rationale for materials with such a hierarchical structure.

Correspondingly, good impedance matching is an important prerequisite for ensuring the occurrence of the above strategies. Smith chart analysis is crucial for understanding the impedance matching characteristics between the material and free space, as detailed in Figure S15. Figure 3m,n, respectively, present 2D impedance matching contour maps for CMX_3@CF_m_/EP and CMX_5@CF_m_/EP, where the remarkably large green regions signify excellent impedance matching. This allows a substantial portion of EMWs to enter the material interior, which is a prerequisite for achieving high electromagnetic absorption capabilities [42, 43]. Furthermore, as depicted in Figure 3o, the impedance matching performance of CMX_3@CF_m_/EP and CMX_5@CF_m_/EP at a thickness of 3.9 mm significantly surpasses that of other component material systems. This favorable impedance matching is attributed to the biominetic hierarchical porous structure of the aerogel, which effectively elongates the propagation path of incident EMWs and significantly increases the probability of wave‐material interaction and subsequent attenuation. Once EMWs propagate into the material, their energy dissipates through various loss mechanisms, quantitatively assessed by the attenuation coefficient (α), as depicted in Figure 3p and Figure S16.

(3) $$\begin{array}{c} \alpha =\frac{\sqrt{2}\pi f}{c}\sqrt{\left(\mu^{\prime \prime }\varepsilon^{\prime \prime }-\mu^{\prime }\varepsilon^{\prime }\right)+\sqrt{{\left(\mu^{\prime }\varepsilon^{\prime }-\mu^{\prime \prime }\varepsilon^{\prime \prime }\right)}^{2}+{\left(\mu^{\prime \prime }\varepsilon^{\prime }+\mu^{\prime }\varepsilon^{\prime \prime }\right)}^{2}}} \end{array}$$

In summary, our comprehensive results unveil the multifaceted and synergistic electromagnetic absorption mechanisms inherent to the CMX@CF_m_ aerogels. Figure 3q provides a conceptual model illustrating these intricate mechanisms. The material's multi‐level hierarchical structure further amplifies absorption by effectively elongating the propagation path of EMWs, while the interface polarization resulting from a large number of carefully designed heterogeneous interfaces, along with the inherent dielectric polarization effect collectively serve as the dominant energy dissipation pathways. Together, these integrated mechanisms orchestrate a broad‐spectrum impedance matching and a high attenuation coefficient, ultimately dictating the material's superior EMW absorption performance. It is worth noting that the strategy of using a carbon framework to load CeO_2_@MXene has further enhanced the nanoscale efficacy.

To directly visualize the intrinsic EMW absorption mechanisms within the CeO_2_/MXene, we conducted detailed electric field distribution simulations using COMSOL Multiphysics (Figure 3r). The results reveal significant electric field enhancement and charge accumulation at the CeO_2_‐MXene interface. Under an alternating electromagnetic field, the accumulated charges at these junctions form numerous dipoles, thereby inducing strong interfacial polarization (Maxwell‐Wagner effect). Localized electric field regions and energy dissipation also occur at the interfaces between layers, indicating that as EMWs propagate through the multilayered structure, their energy is effectively attenuated at each successive interface. Furthermore, the simulations explored interactions from a more microscopic perspective, specifically the electromagnetic response between adjacent CeO_2_ nanoparticles. Electric field distribution maps highlight the enhancement of local electric fields and complex scattering phenomena within the gap spaces and around the periphery of adjacent particles. This observation confirms that even at the nanoparticle level, EMWs undergo multiple reflections and collisions, with each interaction providing additional opportunities for energy dissipation. Visualizations of the electric field (E‐field) distribution (Figure 3s) distinctly reveal significant field localization and enhancement, particularly at the interfaces between the carbon scaffold and the dielectric components. Furthermore, the inherent hierarchical porous structure within the design is observed to promote multiple scattering and reflection events within the material. This effectively elongates the propagation path of EMWs, thereby increasing the probability of their attenuation.

**FIGURE 4:**
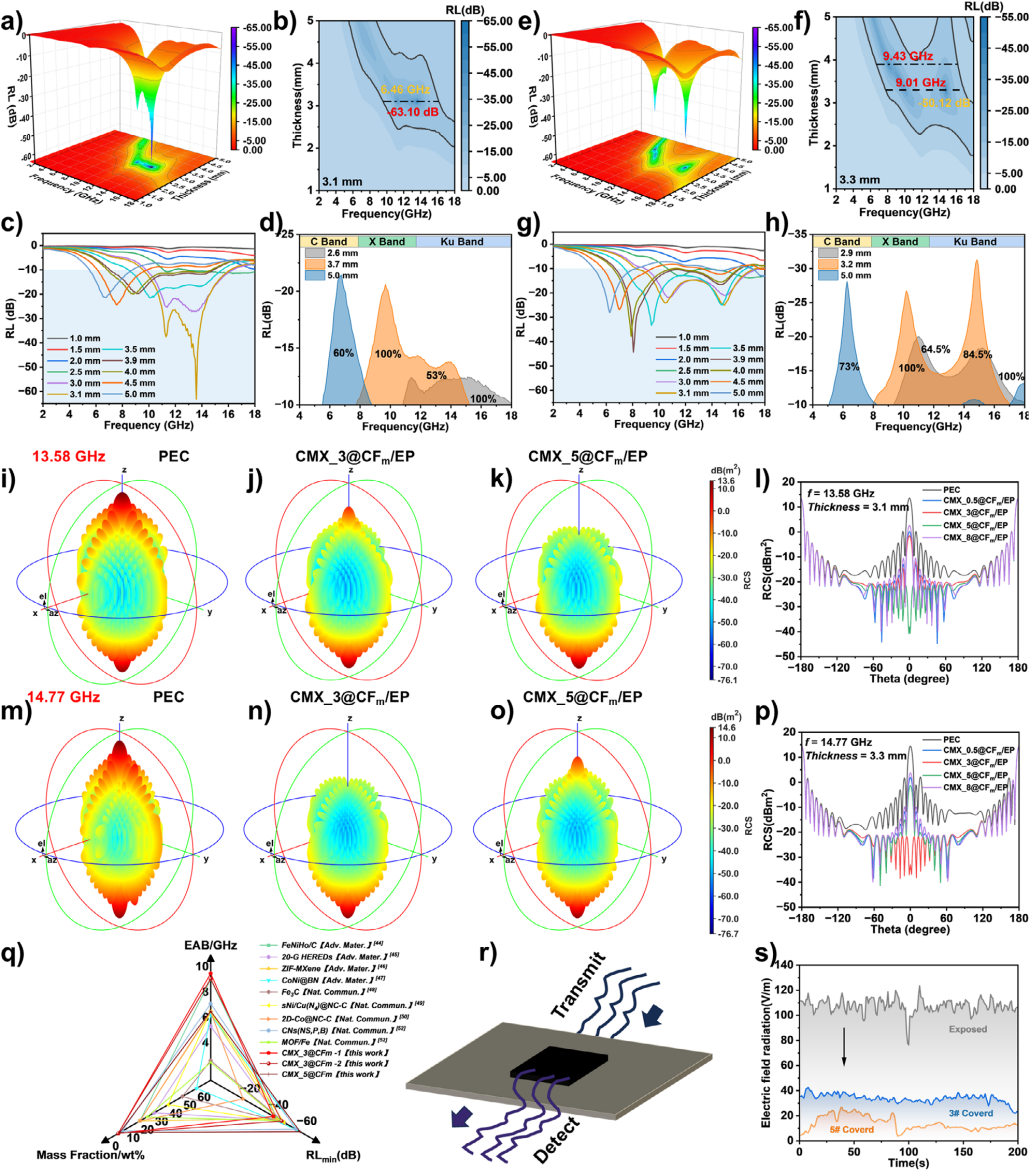
The EMW absorption performance of biomimetic electromagnetic aerogel. The scattering loss spectra of the biomimetic electromagnetic aerogel: (a,b) CMX_5@CF_m_ (e,f) CMX_3@CF_m_; The effective absorption bandwidth of biomimetic electromagnetic aerogel: (c,d) CMX_5@CF_m_ (g,h) CMX_3@CF_m;_ Simulation results of the biomimetic electromagnetic aerogel's RCS at (i‐l)13.58 GHz and (m‐p) 14.77 GHz;(q) Comparison of the performance between biomimetic electromagnetic aerogel and electromagnetic absorption materials in advanced research [44, 45, 46, 47, 48, 49, 50, 51, 52]; (r)Experimental schematic diagram and (s) results of mobile phone electromagnetic radiation protection.

Based on the transmission line theory, the RL for all samples with thicknesses ranging from 1.0 to 5.0 mm was calculated, as presented in Figure S17. Specifically, as shown in Figure 4a–c, the CMX_5@CF_m_/EP exhibited an outstanding RL_min_ value of −63.10 dB at 3.1 mm and 13.58 GHz. This excellent absorption performance was accompanied by a broad EAB of 6.46 GHz, covering the frequency range of 9.59–16.05 GHz. When the thickness was optimized to 4.0 mm, the CMX_5@CF_m_/EP sample achieved its maximum EAB of 7.82 GHz (6.87–14.69 GHz). Even more impressively, as depicted in Figure 4e–g, the CMX_3@CF_m_/EP showed an even wider EAB. At a thickness of 3.9 mm, its maximum EAB reached 9.43 GHz (6.70 – 16.13 GHz). Notably, this remarkable 9.43 GHz effective absorption bandwidth surpasses all previously reported carbon‐based EMW absorbing materials in the literature, a significant feat achieved despite the ultralow aerogel loading of merely 3.74 wt.% in the resin matrix (Table S5). Furthermore, the CMX_3@CF_m_/EP achieved an RL_min_ of ‐50.12 dB at 3.3 mm and 14.77 GHz, while still maintaining an ultra‐wide EAB of 9.01 GHz (7.80 – 16.81 GHz). These results collectively highlight the superior EMW absorption performance of CMX@CF_m_ aerogels across a broad frequency range and at remarkably thin thicknesses. Compared to other representative absorbing materials (Figure 4q and Table S6), the biomimetic absorbing aerogel developed in this study exhibits outstanding performance in both broadband absorption (> 9 GHz) and high loss (< ‐60 dB), far exceeding that of existing similar material systems, while maintaining an exceptionally low filler content of merely 3.74 wt.%. This remarkable combination positions our material at the forefront of carbon‐based EMW absorber research.

The microwave absorption performance across the C‐band (4–8 GHz), X‐band (8–12 GHz), and Ku‐band (12–18 GHz) can be precisely tuned by adjusting the material thickness. As shown in Figure 4d, the CMX_5@CF_m_/EP sample achieved 100% X‐band coverage and 53% Ku‐band coverage at a thickness of 3.7 mm, and 60% C‐band coverage at a thickness of 2.6 mm. Similarly, as depicted in Figure 4h, the CMX_3@CF_m_/EP sample covered 100% of the X‐band and 84.5% of the Ku‐band at a thickness of 3.2 mm, and 73% of the C‐band at a thickness of 2.9 mm. This is attributed to the more pronounced polarization relaxation processes experienced by the designed multi‐level materials at low and mid‐frequencies. Polarization loss, consequently, plays a decisive role in the overall energy dissipation achieved through the intricate multi‐interface construction. By simply adjusting the material's relatively thin thickness, satisfactory multi‐band tunable absorption can be realized, as illustrated in Figure 4b,f. The relevant mechanism analysis can be found in Figures S18–S22.

This broad spectral coverage, coupled with the aerogel's ultralow loading concentration and lightweight nature (density: 0.018–0.117 g/cm^3^), positions them as exceptionally promising candidates for advanced stealth technologies and next‐generation electromagnetic interference shielding applications. To further validate these outstanding EMW absorption characteristics and their practical implications, we conducted comprehensive Radar Cross‐Section (RCS) simulations, depicted in Figure S23. These simulations consistently confirmed the superior EMW absorption performance predicted by our reflection loss measurements (see Figure 4i–p). The RCS analysis provides critical insights into the material's ability to reduce radar detectability of objects, directly affirming the effectiveness of our bio‐inspired hierarchical design in fabricating highly efficient EMW absorbing metamaterials.

Finally, to demonstrate the practical applicability of the hierarchical aerogel in civilian electronics, our prepared resin material was applied to cover a chip within a communication device (Figure 4r). Radiation measurements were subsequently conducted on both the bare chip and the chip covered with our material patch. As illustrated in Figure 4s, the bare chip emitted radiation with an intensity as high as 110 V/m. In stark contrast, our material significantly reduced this radiation intensity to merely approximately 10–40 V/m. This experimental validation underscores the potential of our bio‐inspired structure for real‐world applications in civilian sectors.

Beyond their exceptional EMW absorption capabilities, the as‐prepared aerogels exhibit a suite of other functional properties. Simple compression–recovery tests (Figure S24) demonstrate that the aerogel's robust framework retains considerable elasticity even after the incorporation of nanomaterials. Figures S25 and S26 further illustrates the material's structural integrity, showing minimal deformation under a substantial 100 g load. The hot plate experiment (Figure S27) reveals the aerogel's superior thermal insulation performance: a 10*10*10 mm^3^ sample placed on a hot plate heated to 100–200°C maintained a stable surface temperature below 50°C. Furthermore, combustion experiments (Figures S28 and S29) highlight their structural resilience, with the aerogel's basic framework remaining largely intact even after direct ablation. We also investigated the thermal and thermomechanical properties of the aerogels when filled with epoxy resin. Figure S30 demonstrates that the presence of the aerogel enhances the epoxy resin's room‐temperature thermal conductivity. This is attributed to the uniformly distributed CeO_2_/MXene, coupled with the complete 3D carbon framework, which collectively increase the phonon transport efficiency within the epoxy resin. The nanosheets coating the carbon scaffold provide an extended network for phonon transmission. Moreover, the material exhibits a slight improvement in thermal stability (Figure S31 and Table S7). More notably, Figure S32 andTable S8 show an increase in the glass transition temperature (T_g_​), resulting from the interlocking forces between the well‐impregnated CeO_2_/MXene‐loaded 3D carbon framework and the epoxy resin. This interaction hinders the segmental motion of polymer chains. Simultaneously, the continuous and intact 3D network of the carbon scaffold impedes stress propagation, further contributing to the elevated glass transition temperature. Additional experimental results are detailed in the Supplementary Information.

In summary, the designed bio‐inspired multi‐level hierarchical aerogels possess a range of promising multifunctional characteristics, significantly broadening their potential application domains.

## 3 Conclusions

In conclusion, we successfully developed high‐performance EMW absorbing aerogels through a novel biomimetic strategy inspired by *Melanophila acuminata* beetles. Our design features a multi‐level hierarchical structure integrating a carbon framework with CeO_2_ nanoparticles and Ti_3_C_2_T_x_ MXene nanosheets. The intrinsic hierarchical porous structure enhances absorption by elongating the propagation path and facilitating multiple reflections and scattering events within the aerogel. Meanwhile, the rich heterogeneous interfaces bring about interface polarization, accompanied by excellent dielectric polarization loss, achieving outstanding EMW absorption performance. These bio‐inspired aerogels exhibit remarkable EMW absorption capabilities, achieving an outstanding 9.43 GHz effective absorption bandwidth (6.70–16.13 GHz) at a minimal thickness. Notably, this bandwidth surpasses all previously reported carbon‐based EMW absorbing materials in the literature, a significant feat accomplished with an ultralow aerogel loading of merely 3.74 wt.% in the resin matrix, coupled with the aerogels' lightweight nature (0.018 – 0.117 g/cm^3^).

Ultimately, this work presents a highly promising biomimetic route for developing advanced EMW absorbing materials. The combination of broad spectral coverage, high absorption efficiency, ultralow loading, and lightweight characteristics positions these aerogels as exceptionally promising candidates for cutting‐edge stealth technologies and next‐generation electromagnetic interference shielding applications. Our practical validation, demonstrating a substantial reduction in communication device radiation, underscores their immediate relevance for safeguarding civilian electronics from pervasive electromagnetic pollution.

## 4 Methods

### 4.1 Materials

All the materials used in this work are provided in the Supporting Information.

### 4.2 General

Further details on the DFT calculations, electromagnetic simulations (COMSOL Multiphysics), RCS simulations and electromagnetic radiation detection experiment are provided in the Supporting Information.

Biomimetic hierarchical aerogel carbon scaffolds, designated as CMX@CF_m_, were prepared via an impregnation‐then‐inert gas (Ar) heat treatment method. Their specific formulations and corresponding sample designations are detailed in Table S3.

### 4.3 Preparation of Ti_3_C_2_T_x_ MXene Nanosheet

Ti_3_C_2_T_x_ MXene nanosheets were synthesized by etching Ti_3_AlC_2_ MAX phase using a conventional LiF‐HCl system. Specifically, 1 g of lithium fluoride (LiF) powder was slowly added to 20 mL of 9 M hydrochloric acid (HCl) in a 50 mL polytetrafluoroethylene (PTFE) beaker. The mixture was magnetically stirred for 15 min in an ice‐water bath until LiF completely dissolved. Subsequently, 1 g of Ti_3_AlC_2_ was slowly introduced into the LiF‐HCl solution, and the mixture was continuously stirred for 45 h at 45 ∘C. Following the reaction, the resulting mixture was centrifuged at 3500 r/min and washed with deoxygenated water to remove unreacted acid and impurities. This centrifugation‐washing process was repeated 6–7 times until the supernatant pH approached 7. The obtained precipitate (multilayer MXene) was then dispersed in an appropriate amount of deoxygenated water and sonicated for 1 h under an argon atmosphere to prevent oxidation of the MXene lamellar structure. Finally, the sonicated MXene dispersion was freeze‐dried for 72 h to yield a dry and pure Ti_3_C_2_T_x_ MXene nanosheet powder.

### 4.4 Preparation of CeO_2_/MXene Dispersion

For the dispersion, 542.5 mg of Ce(NO_3_)_3_·6H_2_O powder was added to a beaker containing 40 mL of deionized water and heating at 40°C and stirring for 2 h. Separately, 215.0 mg of Ti_3_C_2_T_x_ MXene powder was added to 40 mL of deionized water and ultrasonically dispersed for 1 h at room temperature. These two dispersions were then slowly mixed, diluted with deionized water, and continuously stirred to ensure uniform dispersion.

### 4.5 Preparation of Biomimetic Hierarchical Aerogels CMX@CF_m_

Melamine foam was cut into 40 mm × 30 mm × 30 mm blocks, rinsed three times each with deionized water and absolute ethanol, and then air‐dried at room temperature for 48 h. The dried melamine foam blocks were immersed in the prepared dispersion, compressed three times to ensure thorough absorption. The foam‐dispersion mixture was then sonicated for 1 h to ensure complete and uniform dispersion of the raw materials within the foam. After sonication, the foam was retrieved and freeze‐dried under vacuum for 72 h to obtain the fully dried precursor foam.

The dried precursor foam was subsequently placed in a tube furnace and subjected to thermal treatment under a 200 mL/min argon (Ar) flow. Prior to heating, the tube furnace was purged with an argon flow at room temperature for 1 h to ensure complete removal of air. The heat treatment involved heating at 350°C for 3 h, followed by heating at 800°C for 3 h. After the heat treatment, the samples were allowed to cool naturally with the furnace to room temperature before being retrieved as CMX@CF_m_.

### 4.6 Preparation of EMW Absorbing Test Samples CMX@CF_m_/EP

CMX@CF_m_/EP EMW absorbing test samples were prepared via a vacuum‐assisted impregnation method. First, epoxy resin and an amine curing agent were mixed at a mass ratio of 100:35 and mechanically stirred until thoroughly homogeneous. This resin‐curing agent mixture was then placed in a vacuum drying oven at 35°C for 15 min under vacuum to remove any air bubbles. Next, the previously prepared CMX@CF_m_ aerogel was immersed into this mixture and allowed to sit for 5 min to ensure complete liquid penetration. With the CMX@CF_m_ fully submerged, the assembly was transferred back to the preheated (35°C) vacuum drying oven and left under vacuum for another 15 min. This step was critical for expelling any remaining bubbles from the system, ensuring complete impregnation of the aerogel by the resin. Subsequently, the drying oven temperature was raised to 80°C, and the mixture was cured for 2 h, yielding the epoxy resin‐filled CMX@CF_m_/EP aerogel.

### 4.7 Characterization

The surface morphology of the sample and microscopic photographs of beetle were obtained by scanning electron microscopy (SEM, JSM7500, JEOL, Japan; TESCAN CLARA, TESCAN, Czech Republic; TESCAN VEGA3, TESCAN, Czech Republic). The crystal structure was analyzed using x‐ray diffraction (XRD, D/MAX 2500, Rigaku, Japan). The low‐angle crystal structure was measured by the small‐angle x‐ray scattering method (SAXS) using a copper target (Xeuss 3.0 HR, Xenocs, France). The chemical composition of the sample was tested using Raman spectroscopy (LabRAM HR Evolution, HORIBA, France), with a laser wavelength of 633 nm. Further chemical composition analysis of the sample was carried out using the x‐ray photoelectron spectroscopy scanning method (XPS, ESCALab220i‐XL, Thermo Fisher Scientific, USA), employing monochromatic Al *K*_α_ radiation with a scanning step of 1.0 eV. The wettability was characterized by measuring the infiltration time of the liquid on the sample surface (OCA25, Dataphysics, Germany). The test liquid was uniformly mixed from epoxy resin and amine curing agent in a mass ratio of 100:35, with air bubbles removed, and the drop size of 4 µL, to investigate the impregnation effect of the epoxy resin on the aerogel. The specific surface area of the sample was measured by the nitrogen adsorption‐desorption method (JW‐BK112, Jingwei Gaobo, China). The electrical conductivity of the sample was tested using the electrochemical impedance spectroscopy (EIS, CHI 660E, CH Instruments, China), with a frequency range of 0.1 to 100,000 Hz and an amplitude of 5 millivolts. For the electromagnetic wave absorption test, the sample was mechanically processed into a ring with an outer diameter of 7 mm, an inner diameter of 3 mm, and a thickness of 2 mm. Its electromagnetic parameters were measured using the coaxial transmission line method, with a frequency range of 2 – 18 GHz (8722ES, Keysight/Agilent, USA). Additional experimental procedures are detailed in the supplementary information.

## Author Contributions

**Zhiwei Liu**: Conceptualization, Data curation, Investigation, Methodology, Writing – original draft, Writing – review & editing. **Dingyu Xu**: Investigation, Methodology, Writing – original draft; **Zhaobo Liu**: Data curation, Methodology; **Haitian Song**: Investigation; **Qianqian Zhang**: Writing – original draft; **Junxiao Zhou**: Methodology; **Xiewen Wen**: Conceptualization, Funding acquisition, Supervision.

## Conflicts of Interest

The authors declare no conflict of interest.

## Supporting information




**Supporting File**: advs76496‐sup‐0001‐SuppMat.docx.

## Data Availability

Data related to this paper may be requested from the authors.
