# Supplementary material for: Modified MXene Aerogel With Broadband Microwave Absorption Inspired by Melanophila Acuminata Beetle
Source: Adv Sci (Weinh). 2026 Jul 13;13(55):e76496. doi: 10.1002/advs.76496 (PMC13359398; doi:10.1002/advs.76496)
Supplement: Supplementary file 1 [file ADVS-13-e76496-s001.docx]

*Supporting Information for*

**Modified MXene aerogel with broadband microwave absorption inspired by Melanophila acuminata beetle**

***Zhiwei Liu^1,2+^, Dingyu Xu^1,2+^, Zhaobo Liu^3^, Haitian Song^4^, Qianqian Zhang^1,2^ & Xiewen Wen^1,2*^***

^1^State Key Laboratory of Ultra-precision Machining Technology, Department of Industrial and Systems Engineering, The Hong Kong Polytechnic University, Kowloon, Hong Kong, SAR, China.

^2^Research Institute for Advanced Manufacturing, Department of Industrial and Systems Engineering, The Hong Kong Polytechnic University, Kowloon, Hong Kong, SAR, China.

^3^School of Materials Science and Engineering, Beihang University, Beijing, 100191, China.

^4^Fujian Academy of Forestry, Fuzhou, 350012, China.

^#^e-mail: xw.wen@polyu.edu.hk

^+^ These two authors contributed equally to this work

**The file includes:**

Supplementary Tables 1 to 10

Supplementary Figures 1 to 32

Supplementary Notes 1 to 5

**Supplementary Table.S1 The stoichiometric ratio of synthesized MXene**

| **Name** | **Peak Binding Energy**  **/eV** | **FWHM**  **/eV** | **Area (Peak)**  **/CPS.eV** | **Atomic percent**  **/%** |
| --- | --- | --- | --- | --- |
| Ti 2p | 455.22 | 3.61 | 3793057 | 29.80 |
| C 1s | 282.10 | 4.78 | 736561.9 | 34.40 |
| O 1s | 529.81 | 4.02 | 938257.1 | 17.26 |
| F 1s | 684.94 | 3.32 | 1304514 | 18.54 |

The XPS analysis performed on Ti₃C₂Tₓ MXene, synthesized via the HCl-LiF etching method, revealed its surface atomic composition. Critically, the surface functionalization of Ti₃C₂Tₓ MXene prepared by this technique was found to be dominated by oxygen-containing and fluorine-containing groups.^[1]^ Consequently, a focused analysis of the atomic percentages of oxygen and fluorine was undertaken, yielding a result closely approximating a 1:1 ratio. This empirical finding subsequently informed the DFT calculations, wherein the Ti₃C₂Tₓ MXene was modeled and defined as Ti₃C₂OF.

**Supplementary Fig.S1 Optimization of heterojunction structure**

The heterojunctions were constructed by modeling a single CeO₂(111) surface with a Ti₃C₂OF surface. Three distinct structural models were built based on different interfacial stacking configurations, as schematically illustrated in Fig. S1.

1. Structure 1 (Fig. S1a): In this configuration, the Ce atoms at the interface, observed from left to right, exhibit sequential alignment characteristics with the Ti₃C₂OF surface. Specifically, the leftmost Ce atom is essentially aligned with the OF termination, the middle Ce atom is slightly offset from the OF termination, and the rightmost Ce atom is positioned between two columns of OF terminations, essentially aligning with the underlying Ti atom (corresponding to the bright green dashed alignment in the schematic).
2. Structure 2 (Fig. S1b): This model presents an alternative interfacial arrangement. The Ce atoms on both side edges (belonging to the same Ce atom) are primarily aligned with the OF columns. Simultaneously, the slightly left-of-center Ce atom is marginally offset from the OF column, while the slightly right-of-center Ce atom is precisely situated between two OF columns, largely aligning with the corresponding Ti atom position (corresponding to the bright green dashed alignment in the schematic).
3. Structure 3 (Fig. S1c): In the third structure, the leftmost Ce atom is aligned with the central position between two OF columns, which also corresponds to a Ti atom position. The central Ce atom is primarily aligned with the OF column, while the rightmost Ce atom is slightly offset from the OF column (corresponding to the bright green dashed alignment in the schematic).

**Supplementary Table.S2 Interface binding energy of heterojunction structure**

| **Structure** | **1** | **2** | **3** |
| --- | --- | --- | --- |
| Interface Binding Energy/eV·Å^-2^ | -0.0280 | -0.0278 | -0.0279 |

Based on the interfacial binding energy data, Structure 1 exhibited the lowest (most negative) interfacial binding energy, indicating the strongest bonding. Consequently, Structure 1 was adopted for all subsequent heterojunction calculations. While the three heterojunctions displayed only minor differences in their interfacial binding energies, this can be attributed to their similar overall alignment patterns. Despite variations in specific stacking, each structure consistently featured one column of Ce atoms aligned with an OF column, another slightly offset from an OF column, and a third positioned between two OF columns. This recurring arrangement suggests a fundamental and stable docking configuration between the CeO₂(111) and Ti₃C₂OF surfaces.

Additionally, for the heterojunction structure obtained in the experiment, we performed XPS testing and compared it with pure MXene. The results are shown in Fig.S2.

**Supplementary Fig.S2 The total XPS spectra of CMX and MXene**

Compared to pure MXene, the Ti 2p in the heterojunction structure shifts towards the higher energy direction, which is consistent with the difference charge density results obtained by our calculation using structure 1 (Fig.1e).

The calculated interface binding energy differences among the three models are extremely small (ΔE < 0.2 meV), which is several orders of magnitude lower than the thermal fluctuation energy at both room temperature (k_BT_≈26 meV) and the experimental synthesis temperature of 800 °C (k_BT_≈93 meV). This energy degeneracy suggests that these configurations are energetically equivalent during the high-temperature growth process. Due to the physical nature of the interface interaction being insensitive to small atomic alignment deviations, these three models exhibit highly consistent electronic structure characteristics, including charge transfer pathways and state density distributions.

Therefore, the chosen model 1 represents the most thermodynamically stable and representative interface structure for subsequent electronic property analysis.

**Supplementary Fig.S3 DOS spectrogram of heterojunction structure**

Fig.S3a illustrates the band gap of the CeO₂(111) material, measured at 2.2166 eV. The corresponding density of states (DOS) plot in Fig.S2b reveals that the conduction band minimum (CBM) is primarily contributed by the Ce 4f orbitals, while the valence band maximum (VBM) is predominantly formed by the O 2p orbitals. For Ti₃C₂OF (Fig.S3c), the Fermi level traverses the band, indicating the material's conductive nature. Its DOS plot (Fig.S3d) shows that the Ti 3d orbitals are the main contributors at the Fermi level. Similarly, the heterojunction (Fig.S3e) also exhibits conductive properties, with the Fermi level crossing the band. As seen in its DOS plot (Fig.S3f), the Ti 3d orbitals remain the primary contributors at the Fermi level for the heterojunction as well.

Further examination of the PDOS revealed significant orbital hybridization at the interface, evidenced by the considerable overlap of Ce 4f, Ti 3d, O 2p, and F 2p electronic states, particularly in the energy range above the Fermi level. This specific orbital overlap signifies the formation of new, hybridized electronic states, likely corresponding to antibonding states, due to the strong covalent interactions between CeO₂ (involving Ce 4f and O 2p orbitals) and the Ti₃C₂OF (involving Ti 3d, O 2p, and F 2p orbitals) at the interface. The involvement of these specific valence orbitals (Ce 4f, Ti 3d, O 2p, F 2p) in this hybridization underscores the complex and robust nature of the interfacial bonding and its direct influence on the electronic landscape relevant to electromagnetic interactions.

**Supplementary Fig.S4 The work functions of CeO_2_ and Ti_3_C_2_OF MXene**

The electrostatic potential curve in Fig.S4 exhibits two distinct steps, representing the vacuum energy levels of the material's upper and lower surfaces, denoted as "upper" and "lower" respectively. These steps arise from applying a dipole correction to surfaces that are not perfectly symmetrical, thereby allowing the different vacuum energy levels of the top and bottom surfaces to become apparent. From the data presented in the figure, it is evident that the work function of the CeO₂(111) lower surface is higher than that of the Ti₃C₂OF upper surface. Consequently, upon forming the heterojunction, electrons are transferred from the Ti₃C₂OF surface to the CeO₂(111) surface.

To further substantiate the authenticity of the charge transfer as obtained from the DFT calculations, we conducted KPFM analysis on the same process-produced CeO_2_ and MXene from this study. The results are shown in Fig.S5.

**Supplementary Fig.S5 KPFM results of (a,b)CeO_2_ and (c,d) MXene**

According to the testing principle, KPFM measures the electric potential difference *V_CPD_* between the needle tip and the sample.

$V_{CPD}=\frac{\Phi_{tip}-\Phi_{sample}}{e}$··································（1）

Among them, $\Phi_{tip}$ represents the work function of the needle tip. As can be seen from Fig.S5, the *V_CPD_* of MXene is higher than that of CeO_2_. Therefore, the work function of CeO_2_ is higher than that of MXene. When forming a heterojunction, electrons flow from MXene to CeO_2_. This is consistent with the calculation results of DFT.

**Supplementary Table.S3 Name and basic parameters of the aerogel samples**

| **Sample** | **Precursor dispersion concentration /mg·mL^-1^** | **Density /g·cm^-3^** | **Volumetric shrinkage rate/%** |
| --- | --- | --- | --- |
| CMX_0@CF_m_ | 0.0 | 0.006 | 2.40 |
| CMX_0.5@CF_m_ | 0.5 | 0.018 | 5.60 |
| CMX_3@CF_m_ | 3.0 | 0.043 | 8.83 |
| CMX_5@CF_m_ | 5.0 | 0.067 | 10.27 |
| CMX_8@CF_m_ | 8.0 | 0.077 | 11.00 |
| CMX_10@CF_m_ | 10.0 | 0.117 | 13.07 |

The precursor dispersion concentration directly corresponds to the theoretical concentration of the synthesized CeO₂/Ti₃C₂Tₓ MXene nanomaterial. This concentration value was then used to back-calculate the required feeding mass of the raw materials. For the aerogel samples, volumetric shrinkage was determined by calculating the ratio between the aerogel sample and the initial melamine foam template.

**Supplementary Fig.S6 The SEM images of biomimetic electromagnetic aerogel：(a,b) MF, (c)CMX_0@CF_m_，(d) CMX_0.5@CF_m_，(e) CMX_3@CF_m_，(f) CMX_5@CF_m_，(g) CMX_8@CF_m_，(h) CMX_10@CF_m_**

To realize a bio-inspired hierarchical architecture, we propose the strategic immobilization of CeO_2_/MXene nanosheets onto a lightweight, three-dimensional (3D) carbonaceous scaffold. Carbon-based frameworks, such as those derived from graphene or carbon nanotubes, offer an ideal substrate owing to their inherent low density, excellent electrical conductivity, large specific surface area, and robust structural integrity, which are conducive to forming interconnected conductive networks and providing ample sites for active absorber deposition.^[2,3]^ This multi-level construct, integrating the nanoscale attributes of CeO_2_ and MXene with the micro architecture of the carbon skeleton, is meticulously designed to generate a profusion of heterogeneous interfaces (e.g., CeO_2_-MXene, MXene-carbon) and a favorably graded impedance profile. Such an arrangement promotes a cascade of EMW loss mechanisms, including enhanced interfacial polarization, dipole relaxation, conductive losses, and extensive multiple scattering, which are crucial for realizing strong and broadband EMW absorption. Furthermore, recent strides in aerogel science and technology offer a versatile platform for fabricating these complex hierarchical structures into ultralight, highly porous materials.^[4]^ Aerogels, distinguished by their exceptionally low densities, high porosity, and vast internal surface areas, can substantially augment EMW absorption by extending the propagation pathways of incident waves, thereby increasing the probability of wave-material interactions and attenuation.^[5]^ The interconnected porous network inherent in aerogels facilitates improved impedance matching with free space, minimizing surface reflection and allowing greater penetration of EMWs into the absorptive matrix.^[6]^ By engineering a CeO_2_/MXene-functionalized carbon aerogel, we aim to synergistically combine the intrinsic EMW absorption prowess of the individual components, further amplified by the bio-inspired hierarchical organization and the unique structural advantages conferred by the aerogel framework. This approach is poised to overcome the performance bottlenecks of conventional absorbers, heralding a new class of lightweight, ultra-broadband EMW absorbing materials.

Fig.S6 shows the SEM images of the MF before and after annealing. Fig.S6(a&b) shows the SEM images of MF at different magnifications. It can be observed that the MF has irregular bubble-like structures, which is in line with our biomimetic strategy. After impregnating the nanomaterial dispersion solution and undergoing annealing, the pore size significantly shrinks, as shown in Fig.S6(c-h). Fig.S4c reveals that in the CMX_0@CF_m_ sample, the carbon skeleton interconnected to form an irregular three-dimensional network structure, thereby creating the aerogel, with the network appearing notably intact. Following immersion in a Ce(NO₃)₃·6H₂O/Ti₃C₂Tₓ MXene precursor dispersion and subsequent high-temperature sintering, particles became attached to the aerogel's skeletal surfaces. As the dispersion concentration increased, the quantity of attached particles progressively grew, as depicted in Fig. S6(d-f). When the dispersion concentration reached 8 mg/mL (Fig. S6g), these attached particles began to coalesce into sheet-like formations, covering the aerogel's cellular pores with a highly non-uniform distribution. Simultaneously, instances of carbon skeleton fracture became apparent. This phenomenon was even more pronounced in the product obtained at a dispersion concentration of 10 mg/mL, as shown in Fig. S6h. In the CMX_10@CF_m_ sample, the carbon skeleton was almost entirely fragmented, with the macroscopic aerogel form being maintained predominantly by large, interconnected particle sheets, while the mechanical properties severely deteriorated. Unbroken three-dimensional carbon frameworks, however, retained their highly porous network structure, with pore sizes largely unchanged.

The observed carbon skeleton fracture is intrinsically linked to the employed synthesis process. The melamine foam, after being fully impregnated with the Ce(NO₃)₃·6H₂O/Ti₃C₂Tₓ MXene dispersion, was freeze-dried and then carbonized at high temperatures in a tube furnace to form the aerogel. During this high-temperature carbonization, the melamine foam skeleton inherently contracts, leading to a reduction in volume. Concurrently with carbonization, the Ce(NO₃)₃·6H₂O/Ti₃C₂Tₓ MXene precursor reacted to generate CeO₂/Ti₃C₂Tₓ MXene nanosheets, which adhered to the skeletal surface and filled the aerogel's cellular pores. Due to the rigid nature of the rare-earth oxide CeO₂ and the transition metal carbide Ti₃C₂Tₓ MXene, these nanosheets impeded the three-dimensional carbon framework's natural contraction during high-temperature carbonization. Consequently, as the precursor dispersion concentration increased, the aerogel's volumetric shrinkage increased, indicating that volume contraction was progressively inhibited, as presented in Table S3. While an appropriate amount of CeO₂/Ti₃C₂Tₓ MXene nanosheets could positively influence the aerogel's volumetric contraction, an excessive increase in content led to extensive nanosheet coalescence and pore filling. This resistance to contraction ultimately caused the carbon skeleton to fracture under the immense forces generated during high-temperature carbonization, manifesting as a collapse of the aerogel's structural integrity. The fragmented carbon skeleton, supported by the large, interconnected CeO₂/Ti₃C₂Tₓ MXene nanosheet layers, could still maintain the aerogel's macroscopic morphology but suffered a drastic decline in mechanical performance. Evidently, such a fractured structure fails to meet the demands of practical applications.

The assembly strategy of these components is crucial for maximizing their synergistic effects. CeO_2_/MXene nanoparticles can be uniformly dispersed within a polymer matrix that subsequently undergoes carbonization to form a porous carbon skeleton. This hierarchical architecture ensures that: (i) CeO_2_ provides distributed polarization centers and broadens the dielectric loss; (ii) MXene forms an efficient conductive network for strong conduction loss and multiple reflections; and (iii) the porous carbon skeleton acts as a lightweight, robust scaffold that optimizes impedance matching and provides additional defect-rich sites.

**Supplementary Fig.S7 The (a)TEM image, (b)SEM image and (c-e)EDS mmapping results of CeO_2_/MXene**

We conducted supplementary experiments to investigate the existence form of CeO_2_ on the surface of MXene, and the results are shown in Fig.S7. We prepared CeO_2_/MXene nanomaterials using the same preparation process and material ratio as CMX_5/MXene, and conducted TEM and SEM tests. In the TEM mode, EDS scanning was performed to confirm the form and state of Ce on the MXene surface. As shown in the figure, the existence form of the sample is that there are a large number of uniformly distributed granular materials (presumed to be CeO_2_) on the surface of the lamellar material (MXene), and the selected area electron diffraction shows a polycrystalline ring. The EDS results show that the distribution of Ce element is consistent with that of Ti element, indicating that the Ce element is uniformly distributed on the MXene surface.

**Supplementary Fig.S8 The XRD spectra of biomimetic electromagnetic aerogel**

As the loading ratio increased, the diffraction peaks of CeO₂ and TiO₂ became progressively stronger, while the broad peak around 20°, indicative of amorphous carbon, almost entirely disappeared. Both the MXene characteristic peaks and CeO₂ characteristic peaks became apparent. Beyond a certain concentration, the increased formation of TiO₂ began to obscure the nearby CeO₂ characteristic peaks. Concurrently, the MXene structure degraded, leading to a reduction in the intensity of its characteristic peaks.

**Supplementary Fig.S9 The Raman spectra of biomimetic electromagnetic aerogel. (a&b)** **Total spectra, I_D_/I_G_ of c)CMX_0@CF_m_，(d) CMX_0.5@CF_m_，(e) CMX_3@CF_m_，(f) CMX_5@CF_m_，(g) CMX_8@CF_m_，(h) CMX_10@CF_m_**

After loading CeO₂/MXene nanosheets onto the aerogel, the I_D_/I_G_ value decreased, which was attributed to the introduction of a more ordered MXene carbon structure. When the loading concentration reaches a certain level, the ordered structure of the carbon framework is disrupted, and the I_D_/I_G_ value rebounds.

**Supplementary Fig.S10 The XPS spectra of the biomimetic electromagnetic aerogel. (a) Total spectra, (b) Ti 2p, (c) C 1s and (d) O 1s spectrum of CMX_5@CF_m_**

After the integrated synthesis, Ti elements representing the Ti_3_C_2_T_x_ MXene were observed in CMX@CF_m_, with the peak position located around 459 eV. Similarly, a group of peaks representing the Ce 3d element appeared within the range of 882 eV to 917 eV, indicating that the Ce element was successfully loaded on the carbon framework. With the increase in the concentration of the precursor dispersion solution, the peaks of the Ce element in the total spectrum gradually strengthened. The Ti 2p could be fitted into four characteristic peaks, with peak positions of 455.27, 456.86, 461.23, and 462.91 eV, corresponding to Ti-C 2p^3/2^, Ti-O 2p^3/2^, Ti-C 2p^1/2^, and Ti-O 2p^1/2^, respectively. The C 1s could be fitted into three peaks, with peak positions of 284.76, 285.93, and 289.13 eV, corresponding to C-C, C-O, and C=O. In the O1s spectrum, a peak at 529.4 eV appeared, representing the Ce-O bond, indicating the successful loading of CeO_2_.

**Supplementary Fig.S11 Results of epoxy resin impregnated biomimetic electromagnetic aerogel**

As shown in Fig.S11, epoxy resin wettability experiments revealed that increasing the loading of nanomaterials led to their aggregation into continuous films within the 3D carbon framework, partially blocking the pores. This, to some extent, hindered the impregnation process of the resin and hardener system.

**Supplementary Fig.S12 (a~c) The SEM images and (d) optical microscope photograph of CMX_5@CF_m_/EP. Among them, Fig.S12(a) shows the image of the junction of the aerogel skeleton and a complete skeleton, and Fig.S12(b) is the enlarged image of the complete skeleton in Fig.S12(a). Fig.S12(c) shows another representative skeleton image from the CMX_5@CF_m_/EP sample. Fig.S12(d) shows the macroscopic image of the CMX_5@CF_m_/EP sample.**

After confirming the feasibility of impregnating and loading aerogels into an epoxy resin and amine-based hardener system, aerogel-filled epoxy resin-matrix were prepared using a vacuum impregnation followed by curing process. Under vacuum conditions, the resin rapidly penetrated the aerogel's internal structure, simultaneously expelling trapped air. Taking the CMX_5@CF_m_/EP sample as an illustrative example, SEM observations were conducted on its polished surface, as shown in Fig. S12. Fig.S12(a-c) are all SEM images of the sample CMX_5@CF_m_/EP, capturing different regions of this sample. These images show information of the same sample (CMX_5@CF_m_/EP) from different perspectives. In Fig.S12(b&c), the general morphology of the aerogel skeleton (the area outlined by the yellow frame) can be observed. In Fig.S12(a), apart from the aerogel skeleton structure seen in Fig.S12(b) (the yellow-framed area), scattered images of the junctions between aerogel skeletons(the area outlined by the red frame) can also be noticed. The boundaries between these skeletons, skeleton junctions and the resin are indistinct, which demonstrates favorable interfacial bonding between the resin and the aerogel skeleton. Moreover, the information presented in the two images differs: Fig.S12(b) prominently displays one intact and clear aerogel skeleton, while Fig.S12(a) shows the skeleton junctions outside the main skeleton regions. Fig.S12(c) shows another representative skeleton image from the CMX_5@CF_m_/EP sample. All the above evidence collectively verifies the excellent combination of resin and aerogel. Furthermore, the resin infiltration within the sample's interior was uniform and thorough, with no discernible air bubbles. Fig. S12(d) presents an optical micrograph of the CMX_5@CF_m_/EP, which reveals a smooth, void-free surface.

**Supplementary Fig.S13 Real part of (a) dielectric constant and (d) magnetic permeability, imaginary part of (b) dielectric constant and (e) magnetic permeability, (c) tangent of dielectric loss angle and (f) tangent of magnetic loss angle of the biomimetic electromagnetic aerogel.**

The real parts of dielectric constant (ɛ') and magnetic permeability (μ') are related to the storage capacity of electromagnetic energy, while the imaginary parts of dielectric constant (ɛ'' ) and magnetic permeability (μ'' ) are associated with energy dissipation and magnetic loss. As shown in Fig.S13, after introducing CeO_2_/Ti_3_C_2_T_x_ MXene nanomaterials, for example, in the biomimetic electromagnetic aerogel, the heterogeneous structure significantly improves. The zero-dimensional, two-dimensional, and three-dimensional interlocking microstructure formed by CeO_2_/Ti_3_C_2_T_x_ MXene and the three-dimensional carbon framework provides a large number of heterogeneous interfaces. Charges accumulate at the interfaces between any two media, and these two media have different dielectric constants, resulting in interface polarization, namely Maxwell-Wagner polarization. The enhancement of the complex dielectric constant of CMX@CF_m_/EP may be related to the multiple interface polarizations between different media. The presence of Ce^3+^ ions/oxygen vacancies in the CeO_2_ nanoparticles deposited on the MXene nanosheets increases the local electronic conductivity. Under an external electromagnetic field, the free electrons on the MXene substrate move directionally and form microwave currents. Increasing the conductivity of the material will lead to an increase in dielectric loss, so the electron transfer between CeO_2_ nanoparticles and MXene nanosheets and the carbon framework in CMX@CF_m_/EP will increase the dielectric loss capacity, bringing it to an appropriate range to improve impedance matching.

When the content of CeO_2_/Ti_3_C_2_T_x_ MXene nanosheets in the CMX@CF_m_/EP system exceeds a certain level, for example, in the CMX_10@CF_m_/EP sample, the complex dielectric constant decreases to the same level as that of epoxy resin. This is because the large area of CeO_2_/Ti_3_C_2_T_x_ MXene nanosheets cross-link and stack, covering the original three-dimensional porous structure of the carbon framework. This results in a significant reduction in the number of interfaces within the system, and the reduction of heterogeneous interfaces leads to a weakened interface polarization behavior. Therefore, the complex dielectric constant decreases. In addition, the closure of pores reduces the number of paths for electromagnetic waves to undergo multiple reflections and scattering within the system, resulting in reduced electromagnetic wave loss.

Furthermore, the dielectric and magnetic performance parameters of CMX@CF_m_/EP change in a downward trend with frequency. The real parts of the complex dielectric constant of CMX_3@CF_m_/EP and CMX_5@CF_m_/EP decrease in the high-frequency field, possibly due to the lag phenomenon. Within the frequency range of 2-18 GHz, due to the small size effect, surface effect, and spin wave excitation, the magnetic permeability of these samples fluctuates to some extent, and since the material is non-magnetic, μ' and μ'' are close to 1 and 0. By calculating the dielectric loss intensity and magnetic loss intensity using the dielectric loss tangent and magnetic loss tangent respectively, the larger the tangent value, the greater the loss intensity. The dielectric loss tangent of CMX_3@CF_m_/EP and CMX_5@CF_m_/EP materials is always greater than the magnetic loss tangent. This also reflects that the attenuation of electromagnetic waves is attributed to dielectric loss.

**Supplementary Fig.S14 The** $\boldsymbol{\varepsilon}^{\boldsymbol{''}}\boldsymbol{-}\boldsymbol{\varepsilon}^{\boldsymbol{'}}$ **curve of biomimetic electromagnetic aerogels**

The Cole-Cole semicircles all exhibit a relatively long "tail", which indicates that the electrical conductance loss mechanism of the biomimetic electromagnetic aerogel is more pronounced. At the same time, the increase in the number of rings also suggests an increase in the polarization relaxation process within the system.

**Supplementary Table.S4 Charge transfer resistance of biomimetic electromagnetic aerogel**

|  | **CMX_0/CF_m_** | **CMX_0.5/CF_m_** | **CMX_3/CF_m_** | **CMX_5/CF_m_** | **CMX_8/CF_m_** |
| --- | --- | --- | --- | --- | --- |
| R_ct_/Ω | 1.98 | 4.35 | 1.81 | 3.34 | 20.09 |

**Supplementary Fig.S15 Smith diagram of biomimetic electromagnetic aerogel**

The center point of the Smith chart represents the normalized input impedance *Z_in_/Z_0_* = 1 + j0, which signifies perfect impedance matching. In this state, the material's input impedance equals that of free space (Z_0_), resulting in zero reflection of electromagnetic waves (Γ= 0) at the material surface, thereby maximizing their entry for attenuation and absorption within the material. Therefore, the performance of an absorbing material largely depends on its impedance matching characteristics: the closer the curve is to the center point of the Smith chart, the better the impedance matching and the more superior the electromagnetic wave absorption performance in that frequency range. Specifically, observing the samples in the figures, the curve for CMX_5/CF_m_@EP has a small segment that is visibly close to the normalized resistance circle R=1.0 and near the center point, suggesting a significant improvement in the impedance matching performance within the corresponding frequency range.

**Supplementary Fig.S16 The impedance matching of (a) EP，(b) CMX_0@CF_m_/EP，(c) CMX_0.5@CF_m_/EP，(d) CMX_3@CF_m_/EP，(e) CMX_5@CF_m_/EP，(f) CMX_8@CF_m_/EP and (g) CMX_10@CF_m_/EP for different thicknesses at 2 - 18 GHz. (h) The impedance matching and (i) attenuation coefficient of biomimetic electromagnetic aerogel at 3.1mm.**

By calculation, we obtained the impedance matching contour plots of the samples at different frequencies and thicknesses, as shown in Fig.S16. It is obvious that the impedance matching performance of pure epoxy resin is poor, with only a few areas having *Z_in_/Z_0_* close to 1 (the green area in the figure). After filling biomimetic electromagnetic aerogel, the impedance matching is optimized, and the areas near 1 increase significantly. The impedance matching performance of CMX_10@CF_m_/E54 material is poor, which is speculated to be due to the excessive filling amount.

By fixing the sample thickness at 3.1 mm (the maximum RL_min_ of the CMX_5@CF_m_/EP sample corresponds to this thickness), the impedance matching values of the samples under different frequencies of electromagnetic waves were calculated, as shown in Fig.S16h. It can be seen that at 3.1 mm, the impedance matching of CMX_3@CF_m_/EP and CMX_5@CF_m_/EP samples is closer to 1 in more frequency bands, and almost all are located in the yellow area in the figure, which indicates that more electromagnetic waves can enter the sample interior at this thickness, which is the prerequisite for the best electromagnetic wave absorption performance. While the impedance matching values of EP, CMX_0@CF_m_/EP, and CMX_10@CF_m_/EP samples are too high, a large amount of electromagnetic waves are reflected at the interface between air and the sample surface and cannot enter the sample interior for dissipation.

The ability of electromagnetic waves to be dissipated after entering the sample is characterized by the attenuation coefficient α. From Fig.S16i, it can be seen that in the 2-18 GHz range, the E54 epoxy resin of CMX_0.5@CF_m_/EP, CMX_3@CF_m_/EP, and CMX_5@CF_m_/EP has a larger attenuation coefficient, indicating that it has a stronger attenuation ability to the incident electromagnetic waves. The increase in attenuation coefficient mainly comes from the various polarization mechanisms mentioned above. In addition, the CeO_2_/Ti_3_C_2_T_x_ MXene nanomaterials in the macroscopic structure maintain the unique layered structure of MXene, and the aerogel presents a three-dimensional open structure within the resin, which has been confirmed by SEM testing and SAXS testing. This enables electromagnetic waves to continuously reflect and scatter after entering the sample, which is conducive to the dissipation of electromagnetic waves. However, although the attenuation coefficients of CMX_0.5@CF_m_/EP and CMX_8@CF_m_/EP samples are relatively consistent, due to the poor impedance matching of CMX_8@CF_m_/EP sample, it cannot effectively dissipate the electromagnetic waves.

**Supplementary Fig.S17 Three-dimensional reflection loss diagram of (a) EP，(b) CMX_0@CF_m_/EP，(c) CMX_0.5@CF_m_/EP，(d) CMX_3@CF_m_/EP，(e) CMX_5@CF_m_/EP，(f) CMX_8@CF_m_/EP and (g) CMX_10@CF_m_/EP. (h)The reflection loss of CMX_5@CF_m_/EP at different thicknesses.**

It should be noted that our work focuses on absorbing materials. The basic principle of these materials is to allow a large amount of electromagnetic waves to enter the interior of the material and then be highly absorbed. However, current electromagnetic shielding materials mainly involve a large number of electromagnetic waves reflecting on the surface and then a small amount of electromagnetic waves entering the interior being absorbed. This is different from the basic principle of absorbing materials. The research on materials with high absorption of electromagnetic waves will play a promoting role in the study of electromagnetic shielding materials from the perspective of the absorption component of electromagnetic shielding.

We compared the performance of the CMX_3@CF_m_ and CMX_5@CF_m_ samples with relatively better performance in the actual characterization at 3mm, as shown in Fig.S17.

Among them, the CeO_2_/MXene content in M_1_, M_2_ and M_3_ was 40 wt%, 45 wt% and 50 wt% respectively of CeO_2_/MXene@C. Through actual testing, the CeO_2_/MXene content in the samples CMX_3@CF_m_ and CMX_5@CF_m_ was 46.19 wt% and 47.62 wt% of the mass fraction of CeO_2_/MXene@CF_m_.

**Supplementary Fig.S18 The simulation and test results of reflection loss at 3mm**

As can be seen from Fig.S18, within the frequency range of 2-18 GHz, the RL variation trend of the CMX@CF_m_ sample is consistent with that of the simulated sample. At around 14 GHz, both show extreme values of RL, while the extreme value highlighted at 11 GHz is related to the quarter-wavelength effect. In terms of the parameter of the effective absorption bandwidth that we focused on, the results of CMX_3@CF_m_ (8.84 GHz) and M_1_ (9.94 GHz) are similar, with an error of about 1 GHz.

The error in material quality fraction and performance is mainly related to the preparation process of the material. In the actual preparation, we adopted the process of mixing and dispersing the precursors and then sintering them together. The dispersion degree of the nanomaterials (CeO_2_/MXene) on the carbon framework (CF_m_) would be affected to some extent, and it was not as uniform as designed in the simulation process. Therefore, there was a loss in the effective absorption bandwidth, and the aggregation of the nanomaterials also caused a small increase in reflection loss. However, the simulation results accurately capture the fundamental EM response trends and the peak frequency positions. This consistency demonstrates that the simplified Voronoi model is a scientifically rigorous tool for predicting the physical mechanisms and guiding the rational design of such hierarchical absorbers.

The relationship between the thickness ($t_{m}$) of the absorbent and the minimal frequency ($f_{m}$) can be expressed by the following equation:^[7]^

$t_{m}=\frac{n\lambda_{m}}{4}=nc\left( 4f_{m}\left| \varepsilon_{r} \right|\left| \mu_{r} \right|^{\frac{1}{2}} \right);n=1,3,5\ldots$·················（2）

where $t_{m}$ is the thickness of the absorbent, which corresponds to the red line, the black diamonds are the matching thickness, $f_{m}$ is the peak frequency, $\lambda_{m}$ is the quarter-wavelength at $f_{m}$, respectively. The frequency dependence of RL at various thicknesses and the frequency dependence of the calculated l/4 thickness for the CMX_3@CF_m_/EP are shown in Fig.S19. The frequency dependence of RL at various thicknesses and the frequency dependence of the calculated l/4 thickness for the CMX_5@CF_m_/EP are shown in Fig.S20.

There is no doubt that the RL_min_ positions are well consistent with the quarter-wavelength match model. Furthermore, as can be seen from the results, both the CMX_3@CF_m_/EP and CMX_5@CF_m_/EP samples exhibit double absorption peaks, and the high-frequency absorption peaks do not shift with the change in thickness. This is caused by the intrinsic absorption resulting from the dielectric loss of the material.

**Supplementary Fig.S19 The frequency dependence of RL at various thicknesses and the frequency dependence of the calculated l/4 thickness for the CMX_3@CF_m_/EP**

**Supplementary Fig.S20 The frequency dependence of RL at various thicknesses and the frequency dependence of the calculated l/4 thickness for the CMX_5@CF_m_/EP**

We have quantified the proportional relationship between the polarization loss and the conduction loss in dielectric loss. The specific method is as follows.

According to the Debye theory, dielectric loss can be expressed as follows:

$\varepsilon^{''}=\varepsilon_{p}^{''}+\varepsilon_{c}^{''}=\left( \varepsilon_{s}-\varepsilon_{\infty} \right)\frac{\omega\tau}{1+\omega^{2}\tau^{2}}+\frac{\sigma}{\omega\varepsilon_{0}}$·····················（3）

Among them, $\varepsilon_{p}^{''}$represents the conduction loss component, $\varepsilon_{c}^{''}$represents the polarization loss component, $\varepsilon_{s}$ is the static dielectric constant, $\varepsilon_{\infty}$is the high-frequency dielectric constant, $\sigma$is the conductivity (measured by a four−probe tester), $\varepsilon_{0}$ is the vacuum dielectric constant, $\omega$is the angular frequency, and $\tau$ is the relaxation time.

Conduction loss is related to the DC conductivity:

$\varepsilon_{c}^{''}=\frac{\sigma}{2\pi f\varepsilon_{0}}$············································（4）

Then the polarization loss is:

$\varepsilon_{p}^{''}=\varepsilon_{measured}^{''}-\varepsilon_{c}^{''}$·······································（5）

Therefore, by using the above formula to quantify the ratio of polarization loss and conduction loss, the results are shown in Fig.S21.

**Supplementary Fig.S21 The polarization loss and conduction loss of CMX_3@CF_m_/EP and CMX_5@CF_m_/EP**

It can be seen that the dielectric loss of the samples is mainly composed of polarization loss. At high frequencies, the conduction loss can be almost ignored, while at low frequencies, there is a small amount of conduction loss that plays a role. This is also consistent with the Cole-Cole graph.

We added CeO_2_@CF_m_/EP and MXene@CF_m_/EP, with the sample content and synthesis route being the same as that of CMX_3@CF_m_/EP, except that the MXene and Ce(NO_3_)_3_·6H_2_O components were removed respectively. Taking into account the advantages of in-situ dispersion and porous structure, we conducted performance tests on samples obtained by simple blending of CeO_2_, MXene, and CF_m_ and then bonded with paraffin. The ratio was still the same as that of CMX_3@CFm, and the mass fraction of paraffin was 50%. The absorption wave performance were plotted, as shown in Fig.S22.

It can be seen from Fig.S22 that the performance of each independent component, namely MXene@CF_m_/EP, CeO_2_@CF_m_/EP and CeO_2_-MXene-CF_m_/wax, is inferior to that of CMX_3@CF_m_/EP. Specifically, MXene@CF_m_/EP achieved a maximum RL_min_ of -27.43dB at 4.8mm (when the frequency was 17.75GHz), and a maximum effective absorption bandwidth of 2.72GHz (when the sample thickness was 5mm). CeO_2_@CF_m_/EP achieved a maximum RL_min_ of -39.04GHz at 2.9mm (when the frequency was 13.50GHz), and a maximum effective absorption bandwidth of 5.78GHz(when the sample thickness was 3.4mm). CeO_2_-MXene-CF_m_/wax achieved a maximum RL_min_ of -15.77 at 3.7mm (when the frequency was 5.42GHz), and simultaneously achieved a maximum effective absorption bandwidth of 1.15GHz.

**Supplementary Fig.S22 The electromagnetic wave absorption properties of (a)MXene@CF_m_/EP, (b)CeO_2_@CF_m_/EP, (c)CeO_2_-MXene-CF_m_/wax and (d)****CMX_3@CF_m_/EP**

For the pure carbon framework, due to its complete network structure, it is unable to achieve excellent impedance matching performance. Electromagnetic waves cannot enter the material interior and most of them are reflected back into the air. This point is elaborated in detail in the text. The same is true for the carbon framework after adding MXene. After adding CeO_2_, the dielectric constant decreases, the impedance matching performance improves, and the presence of oxygen vacancies and the increase of heterogeneous interfaces also enhance the absorption performance. However, for the simple mixed CeO_2_-MXene-CF_m_ without pores, the reduction of pores leads to a deterioration of impedance matching performance. In addition, the specific surface area of the sample significantly decreases, multiple reflection and scattering effects are reduced, the heterogeneous interface is reduced, and the absorption performance deteriorates. The above analysis better supports the source of the excellent absorption performance of CMX_3@CF_m_/EP.

**Supplementary Table.S5 The content of each component in biomimetic electromagnetic aerogels**

| **Sample** | **CF_m_/wt%** | **CeO_2_/Ti_3_C_2_T_x_ MXene/wt%** |
| --- | --- | --- |
| EP | - | -- |
| CMX_0@CF_m_/EP | 0.53 | 0.00 |
| CMX_0.5@CF_m_/EP | 1.58 | 1.05 |
| CMX_3@CF_m_/EP | 3.74 | 3.21 |
| CMX_5@CF_m_/EP | 5.83 | 5.30 |
| CMX_8@CF_m_/EP | 6.47 | 5.94 |
| CMX_10@CF_m_/EP | 9.75 | 9.22 |

**Supplementary Table.S6 Comparison of the electromagnetic wave absorption properties of some of the previously reported related materials**

| Name | Density  /mg·cm^-3^ | EAB  /GHz | RL_min_  /dB | Mass Fraction  /wt% | Thickness  /mm | Ref. |
| --- | --- | --- | --- | --- | --- | --- |
| CNFC/MXene/CoCu@C aerogel | 102.50 | 7.36 | -41.84 | 30.00 | 1.72 | ^[8]^ |
| CS/HAc@MXene aerogel | / | 2.39 | -46.66 | 30.00 | 4.20 | ^[9]^ |
| NRGO/MXene aerogel | 13.00 | 5.00 | ≈-20.00 | 30.00 | 1.93 | ^[10]^ |
| BN/MXene-Fe_3_O_4_ aerogel | 20.00 | 3.85 | -63.69 | / | 2.31 | ^[11]^ |
| Ni/MXene/RGO aerogel | 6.45 | 7.30 | -75.20 | 0.64 | 2.15 | ^[12]^ |
| MXene/polyimide aerogel | 72.00 | 1.04 | -64.70 | / | 5.24 | ^[13]^ |
| CS-GA-MXene aerogel | 37.00 | 6.40 | -51.48 | 32.70 | 4.30 | ^[14]^ |
| BNNS/CS-GO aerogel | 14.70 | 9.44 | -35.00 | / | 3.20 | ^[15]^ |
| LAS/N-GF+rGO aerogel | / | 8.34 | -47.98 | 10.00 | 4.50 | ^[16]^ |
| Fe_3_O_4_/Fe/C/rGO aerogel | 2.90 | 5.76 | ≈-30.00 | 5.00 | 2.30 | ^[17]^ |
| CoS/Co_9_S_8_/Co_3_S_4_/rGO aerogels | / | 6.16 | -33.40 | 3.00 | 2.20 | ^[18]^ |
| CuCo_2_S_4_@EG | / | 4.14 | -72.28 | 7.00 | 1.40 | ^[19]^ |
| Fe_3_C | / | 2.55 | -52.09 | 50.00 | 2.90 | ^[20]^ |
| sNi/Cu(N_4_)@NC-1 | / | 6.44 | -51.70 | 40.00 | 2.10 | ^[21]^ |
| 2D-Co@NC-C | / | 6.41 | -22.87 | 20.00 | 1.82 | ^[22]^ |
| CNs(NS,P,B) | / | 7.05 | -60.00 | 7.50 | 2.60 | ^[23]^ |
| Zn_3_Cu_1_-HHTP | / | 3.70 | -81.62 | 50.00 | 2.95 | ^[24]^ |
| ZIF-MXene | / | 6.32 | -47.35 | 20.00 | 2.50 | ^[25]^ |
| CoNi@BN | / | 2.40 | -49.90 | 25.00 | 4.40 | ^[26]^ |
| MOF/Fe | / | 6.16 | -61.00 | 60.00 | 1.78 | ^[27]^ |
| CMX_3@CF_m_ | 4.30 | 9.43 | -44.33 | 3.74 | 3.90 | this work |
| CMX_3@CF_m_/EP-2 | 4.30 | 9.01 | -50.12 | 3.74 | 3.30 | this work |
| CMX_5@CF_m_/EP-1 | 6.70 | 6.46 | -63.10 | 5.83 | 3.10 | this work |

**Supplementary Fig.S23 Three-dimensional RCS diagrams of (a) PEC，(b) CMX_0@CF_m_/EP，(c) CMX_0.5@CF_m_/EP，(d) CMX_3@CF_m_/EP，(e) CMX_5@CF_m_/EP，(f) CMX_8@CF_m_/EP and (g) CMX_10@CF_m_/EP at 13.58 GHz and 3.1 mm. (h)Two-dimensional RCS diagrams of CMX@CF_m_/EP. Three-dimensional RCS diagrams of (i) PEC，(j) CMX_0@CF_m_/EP，(k) CMX_0.5@CF_m_/EP，(l) CMX_3@CF_m_/EP，(m) CMX_5@CF_m_/EP，(n) CMX_8@CF_m_/EP and (o) CMX_10@CF_m_/EP at 14.77 GHz and 3.3 mm. (p)Two-dimensional RCS diagrams of CMX@CF_m_/EP.**

At 13.58 GHz and 3.1 mm, the RL_min_ value of CMX_5@CF_m_/EP reaches its peak. At 14.77 GHz and 3.3 mm, the RL_min_ value of CMX_3@CF_m_/EP also reaches its peak. For the RCS of the material under different incident angles of electromagnetic waves, both impedance matching and 1/4 wavelength interference have changed. Specifically, for oblique incidence, it is necessary to distinguish between vertical polarization TE waves and parallel polarization TM waves. For TE waves,

$Z_{in(TE)}=Z_{0}\sqrt{\frac{\mu_{r}}{\varepsilon_{r}-{sin}^{2}\theta}}$··································（6）

For TM wave,

$Z_{in(TM)}=Z_{0}\frac{\sqrt{\varepsilon_{r}\mu_{r}-{sin}^{2}\theta}}{\varepsilon_{r}}$··································（7）

Impedance matching is related to the electromagnetic parameters of the material, namely $\varepsilon_{r}\mu_{r}$ and the incident angle 𝜃. When 𝜃 changes, the effective input impedance $Z_{in}$ will undergo nonlinear shifts. When the electromagnetic wave is incident obliquely, according to Snell's law, the propagation path of the wave within the material will be deflected, and the phase cancellation condition will change:

$t_{m}=\frac{n\lambda}{4\sqrt{\varepsilon_{r}\mu_{r}-{sin}^{2}\theta}}$··································（8）

When the thickness $t_{m}$ is fixed, the degree of phase cancellation varies with the electromagnetic parameter $\varepsilon_{r}\mu_{r}$ of the material, as well as the incident angle 𝜃. Therefore, for different materials, the trends of impedance matching and phase cancellation with respect to the incident angle 𝜃 are different, which leads to variations in the RCS trends.

**Supplementary Fig.S24 Simple compression and rebound test procedures of (a~c) CMX_0@CF_m_, (d~f) CMX_5@CF_m_ and (g~i) CMX_10@CF_m_ samples.**

The complete three-dimensional carbon framework assembled into an aerogel exhibits excellent compressive recovery elasticity. When a small amount of CeO_2_/MXene nanomaterials are incorporated, the nanosheets adhere to the surface of the carbon framework and have almost no effect on the compressive recovery performance of the aerogel. However, when the doping amount is increased to a certain extent, due to the large number of CeO_2_/MXene nanosheets blocking the contraction of the aerogel structure during the annealing process, the carbon framework breaks, destroying the structural integrity of the aerogel and causing it to lose its compressive recovery performance. As shown in Fig.S24, (a~c), (d~f), and (g~i) represent the simple compression recovery experiments of samples CMX_0@CF_m_, CMX_5@CF_m_, and CMX_10@CF_m_, respectively. It can be seen that the CMX_0@CF_m_ sample can restore its original state after compression, the CMX_5@CF_m_ sample slightly deforms, while the CMX_10@CF_m_ sample is completely broken.

**Supplementary Fig.S25 Load-bearing demonstration of CMX_5@CF_m_**

A 100g weight was placed on a 20mm × 20mm × 16mm CMX_5@CF_m_, and the thickness of the aerogel remained unchanged.

We conducted a cyclic compression performance test on the CMX_5@CF_m_ sample, as shown in Fig.S26.

**Supplementary Fig.S26 Cyclic compression performance test of the CMX_5@CF_m_**

The addition of CMX has caused the framework of CF_m_ to lose some of its elasticity, resulting in the inability of the deformation to fully recover after compression and unloading.

**Supplementary Fig.S27 Thermal plate heat conduction experiment of CMX_5@CF_m_**

Fig.S27(a&b), (c&d) and (e&f) show the temperature conditions on the upper surface and the middle area of the 10mm × 10mm × 10mm CMX_5@CF_m_ when it reaches stability on a heating plate at 100℃, 160℃, and 220℃, respectively. It can be seen that the temperature remained stable at a temperature slightly above room temperature, indicating that the aerogel has a good heat insulation effect.

**Supplementary Fig.S28 Combustion experiment of CMX_5@CF_m_**

CMX_5@CFm still retains most of its original shape after combustion.

We conducted TG-FTIR tests on the CMX_5@CF_m_ samples, and the results are shown in Fig.S29.

The samples mainly undergo thermal degradation within the temperature range of 400 to 600℃, and the final weight loss always remains at 50%. The infrared curve analyzed at 500℃ shows a sharp double peak near 2350 cm^-1^, which is the characteristic peak of the asymmetric stretching vibration of CO_2_ molecules. The characteristic peak in the low wavenumber region corresponds to the peak at 2350 cm^-1^, belonging to the bending vibration mode of CO_2_. This further confirms the generation of a large amount of CO_2_. The weak multiple peaks (sawtooth-like) appearing in the 3500~3800 cm^-1^ region represent the O-H stretching vibration of gaseous water molecules.

**Supplementary Fig.S29 TG-FTIR test of CMX_5@CF_m_: (a) TG curve, (b) 3D and (c) 2D curves of FTIR, (d) FTIR curve at 500℃**

**Supplementary Fig.S30** **(a) The thermal conductivity at room temperature and (b&c) infrared thermal imaging photos of CMX@CF_m_/EP**

Fig.S30(a) shows the thermal conductivity of the biomimetic electromagnetic aerogel after filling with epoxy resin at room temperature. It can be seen that the room-temperature thermal conductivity of pure epoxy resin is 0.21 W·m^-1^·K^-1^. After filling with the biomimetic electromagnetic aerogel, the thermal conductivity slightly increases. The room-temperature thermal conductivities of CMX_3@CF_m_/EP and CMX_5@CF_m_/EP are more significantly improved, reaching 0.34 and 0.38 W·m^-1^·K^-1^ respectively, which are 1.6 and 1.8 times higher than that of pure epoxy resin. This is because the uniformly distributed CeO_2_/Ti_3_C_2_T_x_ MXene, combined with the complete three-dimensional carbon framework structure, increases the phonon transmission efficiency within the epoxy resin, and the nanosheets coated on the carbon framework surface provide a phonon transmission network. When the content of CeO_2_/Ti_3_C_2_T_x_ MXene nanosheets is further increased, although the nanosheets cross-link into a network, which increases the phonon transmission path, the large area of the nanosheets causing the fragmentation of the carbon framework greatly affects the phonon transmission efficiency, thus the thermal conductivity decreases. Fig.S30(b) and (c) show the infrared thermal imaging photos of CMX@CF_m_/EP on a 200 ℃ hot plate. Fig.S30(b) from left to right and from top to bottom are EP, CMX_0@CF_m_/EP, CMX_0.5@CF_m_/EP, and CMX_3@CF_m_/EP, Fig.S30(c) from left to right and from top to bottom are EP, CMX_5@CF_m_/EP, CMX_8@CF_m_/EP, and CMX_10@CF_m_/EP. The infrared thermal imaging photos can more intuitively observe the improvement of thermal conductivity.

**Supplementary Fig.S31 Thermal gravimetric curve of CMX@CF_m_/EP**

As can be seen from Fig.S31, all the CMX@CF_m_/EP samples experienced weight loss around 300 ℃. The weight loss slowed down when the temperature reached around 400 ℃, and the thermogravimetric curve entered the plateau region. When the temperature further increased to around 520 ℃, the mass decreased rapidly until the samples completely decomposed. The weight loss around 300 ℃ was caused by the epoxy resin, and there was little difference in the initial weight loss temperature among the groups of samples. However, when the thermogravimetric curve entered the plateau region, the CMX@CF_m_/EP samples showed significant changes. Within the range of 400 to 500 ℃, the weight loss rates of EP and CMX_0@CF_m_/EP samples were significantly higher than those of other samples. This was because the CeO_2_/Ti_3_C_2_T_x_ MXene nanomaterials had high thermal stability within 500 ℃, and the presence of the nanomaterials after being filled into the epoxy resin enhanced the thermal stability. When the temperature was raised above 600 ℃, the samples completely decomposed.

**Supplementary Table.S7 Heat resistance factor of CMX@CF_m_/EP**

| **Sample** | **Typical temperature/℃** | | **Heat resistance factor/℃** |
| --- | --- | --- | --- |
|  | **T_5_** | **T_30_** |  |
| EP | 348.9 | 373.9 | 178.3 |
| CMX_0@CF_m_/EP | 345.4 | 374.4 | 177.8 |
| CMX_0.5@CF_m_/EP | 353.8 | 373.8 | 179.2 |
| CMX_3@CF_m_/EP | 347.8 | 371.8 | 177.5 |
| CMX_5@CF_m_/EP | 347.6 | 373.5 | 177.9 |
| CMX_8@CF_m_/EP | 347.1 | 373.6 | 177.9 |
| CMX_10@CF_m_/EP | 350.6 | 373.6 | 178.6 |

Obviously, since CMX@CF_m_/EP has already experienced significant weight loss around 300 ℃, the improvement in thermal stability from the filling of CMX@CF_m_ at 400-500 ℃ cannot meet the requirements of practical applications. To further investigate the thermal resistance of CMX@CF_m_/EP, we calculated the thermal resistance index (T_Heat-resistance index_) for all samples according to formula 1, as shown in Table S7. The calculation formula is as follows:

*T_Heat-resistance index_*=0.49×[*T_5_*+0.6×(*T_30_*-*T_5_*)] ·························（9）

Where T_5_ is the temperature when the sample's mass loss is 5%, and T_30_ is the temperature when the sample's mass loss is 30%.
From the Table.S7, it can be seen that the T_5_ of the CMX_0@CF_m_/EP sample slightly decreased, which may be due to the complete three-dimensional framework structure increasing the heat conduction efficiency within the resin, allowing the external temperature to be distributed more quickly and uniformly within the resin, resulting in a lower resin decomposition temperature. After adding the biomimetic electromagnetic aerogel filler, the T_5_ of the CMX@CF_m_/EP sample slightly increased, which is consistent with the conclusion drawn earlier that CeO_2_/MXene nanomaterials filled into epoxy resin enhance the thermal stability of the composite material. However, due to the low filler content, the increase in T_5_ is not significant, only reaching the same temperature as pure epoxy resin. For the T_30_ characteristic temperature, the influence of adding CMX@CF_m_/EP on epoxy resin is even less obvious. The calculated thermal resistance index of CMX@CF_m_/EP is not significantly different, indicating that the filling of CMX@CF_m_ has no significant effect on the thermal weight loss of epoxy resin at 300 ℃, while the thermal stability of the composite material slightly improves between 400-500 ℃.

**Supplementary Fig.S32 (a) Energy storage modulus and (b) loss factor of CMX@CF_m_/EP**

The study of dynamic mechanical properties can effectively examine the bonding situation between epoxy resin and CMX@CF_m_. At the same time, the characterization of thermal mechanical properties can effectively reflect the thermal mechanical properties of the material during its actual application. The magnitude of the storage modulus and loss factor to a certain extent determines the upper limit of the actual usage temperature of the material. As shown in Fig.S32, the decrease in storage modulus and the peak of loss factor are caused by the softening of epoxy resin at high temperatures and the movement of molecular chain segments. During the process of gradually increasing temperature, the epoxy resin transforms from the glassy state to the high elastic state, and the storage modulus drops sharply. The characteristic temperature point at which this transformation occurs is called the glass transition temperature (T_g_). Usually, the peak temperature of loss factor tanδ is defined as the glass transition temperature. In addition, the storage modulus can to some extent reflect the modulus, hardness and load capacity of the material.

**Supplementary Table.S8 Dynamic thermomechanical properties of CMX@CF_m_/EP**

| **Sample** | **Storage modulus [40 ℃]/GPa** | **T_g_/℃** |
| --- | --- | --- |
| EP | 3.243 | 101.8 |
| CMX_0/CF_m_/EP | 2.799 | 108.8 |
| CMX_0.5/CF_m_/EP | 2.739 | 107.4 |
| CMX_3/CF_m_/EP | 3.078 | 105.3 |
| CMX_5/CF_m_/EP | 2.980 | 106.8 |
| CMX_8/CF_m_/EP | 2.880 | 106.7 |
| CMX_10/CF_m_/EP | 3.190 | 104.7 |

As can be seen from Table S8, after filling with CMX@CF_m_, the storage modulus before glass transition decreased slightly, but remained basically at around 3 GPa. This is because the carbon framework itself is relatively soft and has a loose structure, while the molecular weight of the epoxy resin is highly cross-linked and has a high modulus. After filling with CMX@CF_m_, the glass transition temperature significantly increased, which can be clearly observed in Fig.S32 (b). This is because the well-impregnated CMX@CF_m_ and the epoxy resin have developed a frictional force, hindering the segmental movement of the polymer chains, and thus increasing the glass transition temperature. At the same time, the complete and continuous three-dimensional network structure of the carbon framework hinders the propagation of stress, which to some extent enhances the glass transition temperature. It is worth noting that compared with other samples, the glass transition temperature of CMX_10@CF_m_/EP is lower, indicating that the connected sheet-like CeO_2_/Ti_3_C_2_T_x_ MXene nanomaterials are to some extent unfavorable for the resin to fully impregnate the three-dimensional carbon framework.

**Supplementary Note 1. Materials:**

Ti₃AlC₂ MAX phase (400 mesh, 98%) was purchased from Jilin 11 Technology Co., Ltd., China. Lithium fluoride (LiF, 99%) was acquired from Shanghai Aladdin Biochemical Technology Co., Ltd., China. Concentrated hydrochloric acid (HCl, 36-38%) was obtained from Beijing Modern Oriental Technology Development Co., Ltd., China. Cerium(III) nitrate hexahydrate (Ce(NO₃)₃·6H₂O, 99.95%) was purchased from Shanghai Macklin Biochemical Technology Co., Ltd., China. Melamine foam (MF, 9.22 g·cm⁻¹) was acquired from Guangzhou Lüyuan Technology Co., Ltd., China. Epoxy resin and amine curing agent were obtained from Shenyang Dongnan Chemical Research Institute, China. Anhydrous ethanol (CH₃CH₂OH, analytical reagent grade) was purchased from Sinopharm Chemical Reagent Co., Ltd., China.

**Supporting Note 2. DFT calculations:**

Our density functional theory (DFT) calculations were conducted using the Quantum Espresso (QE) package, a first-principles computational tool. For the exchange-correlation functional, we employed the Perdew-Burke-Ernzerhof (PBE) formulation within the generalized gradient approximation (GGA). Projected augmented wave (PAW) pseudopotentials were used to describe the interactions between ionic cores and valence electrons. The valence electron wavefunctions were expanded in a plane-wave basis set with a kinetic energy cutoff of 450 eV, while the cutoff for charge density and potential was set to 4500 eV. To manage partial occupancies of Kohn−Sham orbitals, the Gaussian smearing method was utilized with a width of 0.05 eV. For geometry and lattice parameter optimizations, Brillouin zone integration was performed using a 2×2×1 Γ-centered k-point mesh. The self-consistent field (SCF) iterations were converged to an energy threshold of 10⁻⁵ eV. Structural equilibrium was achieved by optimizing geometries and lattice constants until the forces on individual atoms were less than 0.02 eV/Å. Dispersion forces were accounted for via the DFT-D3 method with Grimme's empirical correction scheme. Magnetic systems were described by implementing a spin-polarized approach. To accurately capture the strong correlation effects of transition metals in the system, all structural optimizations and electronic structure calculations incorporated the spin-dependent GGA+U approach (Hubbard U correction). The effective U_eff_ values applied were 4.0 eV for Ti atoms and 5.0 eV for Ce atoms.

**Supporting Note 3. COMSOL electromagnetic simulation:**

To investigate the electromagnetic properties, the electric field distribution within the material was simulated using the Electromagnetic Waves, Frequency Domain (ewfd) interface in COMSOL Multiphysics under a unit excitation field. A two-port electric field excitation was employed, and the simulated geometric dimensions were referenced from scanning electron microscopy (SEM) images.

Specifically, for the CeO₂/MXene nanomaterial, a "particle-film" electromagnetic model was developed. This model comprised a layer of cerium dioxide (CeO₂) particles, 20 nanometers in diameter, situated on a 100-micrometer-thick Ti₃C₂Tₓ MXene film. The fundamental unit cell was defined by nine CeO₂ particles uniformly distributed on the MXene film, which was then periodically replicated for three cycles along the z-axis direction. The refractive indices of CeO₂ and MXene (see **Table S9**), serving as inputs for this model, were experimentally determined using an Abbe refractometer (BM-2WAJ, BM Microscope, China).

**Supplementary Table.S9 The refractive indices of CeO₂ and MXene**

| **Component** | **Material** | **Refractive index (20℃)** |
| --- | --- | --- |
| Particle | CeO_2_ | 1.3637 |
| Film | MXene | 1.6286 |

For the aerogel material, a periodic model featuring a uniform, Voronoi-like skeletal structure was constructed in Abaqus. This structure was defined by a unit cell with a side length of 25 micrometers and a skeleton radius of 1 micrometer. The electromagnetic properties of these models were defined by their complex relative permittivity and complex relative permeability. The material properties were based on a powder of CeO₂/MXene and carbon powder mixed in a specific proportion. To better simulate the carbon backbone, we opted to use powdered carbonaceous aerogel as the carbon powder. The electrical conductivity ($\sigma$) of powder was measured using a powder resistivity tester (ST2742B, Lattice Electronics, China). The complex relative permittivity and complex relative permeability were determined using a Vector Network Analyzer (VNA) via the coaxial line method. To comply with VNA specifications for toroidal samples, the powder was uniformly mixed with paraffin wax and pressed into a toroidal shape. Subsequently, the measurement data from the paraffin-powder mixture was processed using the Maxwell-Garnett effective medium theory to extract the intrinsic electromagnetic parameters of the pure powder. These calculated values were then input into the simulation model.

The Maxwell-Garnett theory is applicable to situations where one material is dispersed within another continuous matrix to form a mixture. In the aforementioned coaxial test sample, paraffin wax serves as the matrix, and the mixed powder of CeO₂/MXene and carbon acts as the dispersed phase.

The relevant formulas are as follows:

Maxwell-Garnett Equation (for permittivity):

$\frac{\varepsilon_{eff}-\varepsilon_{m}}{\varepsilon_{eff}+2\varepsilon_{m}}=f_{d}\frac{\varepsilon_{d}-\varepsilon_{m}}{\varepsilon_{d}+2\varepsilon_{m}}$·······························（10）

Maxwell-Garnett Equation (for permeability):

$\frac{\mu_{eff}-\mu_{m}}{\mu_{eff}+2\mu_{m}}=f_{d}\frac{\mu_{d}-\mu_{m}}{\mu_{d}+2\mu_{m}}$·······························（11）

Where:

- $\varepsilon_{eff}$ and $\mu_{eff}$ represent the **complex relative permittivity** and **complex relative permeability** of the **mixture**, respectively.
- $\varepsilon_{m}$ and $\mu_{m}$ represent the **complex relative permittivity** and **complex relative permeability** of the **matrix** (paraffin wax), respectively.
- $\varepsilon_{d}$ and $\mu_{d}$ represent the **complex relative permittivity** and **complex relative permeability** of the **dispersed phase** (CeO₂/MXene and carbon powder mixture), respectively.
- $f_{d}$ is the **volume fraction** of the dispersed phase.

The volume fraction of the dispersed phase ($f_{d}$) can be calculated from mass fractions and densities using the following formula:

$f_{d}=\frac{\omega_{d}/\rho_{d}}{\omega_{d}/\rho_{d}+\omega_{m}/\rho_{m}}$··································（12）

Where:

- $\omega_{d}$ is the **mass fraction** of the dispersed phase.
- $\omega_{m}$ is the **mass fraction** of the matrix.
- $\rho_{d}$ is the **density** of the dispersed phase.
- $\rho_{m}$ is the **density** of the matrix.

Relevant parameters are detailed in **Table S10**.

**Supplementary Table.S10 Simulation parameters of aerogel**

| **Sample** | **[**$\frac{\boldsymbol{CeO}_{\boldsymbol{2}}}{\boldsymbol{CeO}_{\boldsymbol{2}}\boldsymbol{+MXene}}$**]**  **/wt%** | **[**$\frac{\boldsymbol{CeO}_{\boldsymbol{2}}\boldsymbol{+MXene}}{\boldsymbol{CeO}_{\boldsymbol{2}}\boldsymbol{+MXene+C}}$**]**  **/wt%** | $\boldsymbol{\sigma}_{\boldsymbol{d}}$  **/S·cm^-1^** | $\boldsymbol{\rho}_{\boldsymbol{d}}$  **/g·cm^-3^** | $\boldsymbol{\omega}_{\boldsymbol{d}}$  **/wt%** | $\boldsymbol{\rho}_{\boldsymbol{m}}$  **/g·cm^-3^** |
| --- | --- | --- | --- | --- | --- | --- |
| M_1_ | 50 | 40 | 0.37940 | 1.086 | 50 | 0.900 |
| M_2_ |  | 45 | 0.11903 | 0.970 | 50 |  |
| M_3_ |  | 50 | 0.14399 | 1.408 | 80 |  |

A material's EM response can be conceptualized as a transmission line. The S-parameters (S11 and S21) from our COMSOL simulations were converted into equivalent ABCD parameters for the unit cell. The ABCD matrix relates the voltage (V) and current (I) at the input port of a two-port network to those at its output port:

$\left( \begin{matrix} V_{1} \\ I_{1} \end{matrix} \right)=(\begin{matrix} A & B \\ C & D \end{matrix})(\begin{matrix} V_{2} \\ I_{2} \end{matrix})$ ··································（13）

For a reciprocal network in a system with characteristic impedance *Z_0_*, the relationship between S-parameters and ABCD parameters is given by:

$A=\frac{\left( 1+S_{11} \right)\left( 1-S_{22} \right)+S_{12}S_{21}}{2S_{21}}$································（14）

$B=Z_{0}\frac{\left( 1+S_{11} \right)\left( 1+S_{22} \right)-S_{12}S_{21}}{2S_{21}}$································（15）

$C=\frac{\left( 1-S_{11} \right)\left( 1-S_{22} \right)-S_{12}S_{21}}{2Z_{0}S_{21}}$································（16）

$D=\frac{\left( 1-S_{11} \right)\left( 1+S_{22} \right)+S_{12}S_{21}}{2S_{21}}$································（17）

The power of the ABCD matrix lies in its ability to cascade multiple identical layers. If a single unit cell has an ABCD matrix Munit, a stack of N such unit cells (representing a material of thickness *N×t_unit_*, where *t_unit_* is the effective thickness of the simulated unit cell) is represented by multiplying their individual matrices: $M_{total}=M_{unit}^{N}$ . This matrix multiplication inherently accounts for all internal reflections and transmissions between stacked layers, providing the overall transfer characteristics of the combined material.

Once the total ABCD matrix (*M_total_*) for the 3 mm thickness was obtained, it was converted back to the overall S-parameters of the macroscopic sample. Specifically, the reflection coefficient (S_11_) for the full thickness is given by:

$S_{11}=\frac{A+\frac{B}{Z_{0}}-CZ_{0}-D}{A-\frac{B}{Z_{0}}+CZ_{0}+D}$··································（18）

**Supporting Note 4. RCS electromagnetic simulation:**

To evaluate the Radar Cross Section (RCS) performance of the samples at specific frequencies, numerical simulations were conducted using the electromagnetic simulation software, CST Studio Suite. The core structure of the simulation model consists of a Perfect Electric Conductor (PEC) substrate covered by the sample under test. The dimensions of the PEC substrate were set to be electrically large to eliminate edge diffraction effects, with its thickness uniformly set to 1.0 mm. The sample layer was placed on top of the PEC substrate, and its material properties—complex permittivity ($\varepsilon_{r}$) and complex permeability ($\mu_{r}$)—were defined based on experimentally measured values. Models were established for two specific configurations: a 3.1 mm thick sample to be analyzed at 13.58 GHz, and a 3.3 mm thick sample at 14.77 GHz. In the simulation, a plane wave was used as the excitation source, illuminating the sample surface at normal incidence. The boundary conditions were set to open (PML) to simulate an infinite free-space environment. A far-field monitor was configured to accurately calculate the monostatic RCS value at these specified frequencies. By running the solver for each configuration, the RCS performance data for each sample at its corresponding designated frequency was ultimately obtained.

**Supporting Note 5. Additional experimental procedures:**

Using sapphire as the reference, the specific thermal capacity ($Cp$) of the samples at room temperature (DSC-3, NETZSCH, Germany) and the thermal diffusivity ($\alpha$, LFA 467 HT Hyper Flash, NETZSCH, Germany) were determined. During the thermal diffusivity test, the surface of the sample needed to be treated with carbon spraying to increase the absorbance. The calculation of thermal conductivity requires the use of the following formula:

$\lambda=\rho\times Cp\times\alpha$································（19）

$\rho$ represents density, with the unit of g/cm³, $Cp$ represents specific heat, with the unit of J/(g·K), and $\alpha$ represents thermal diffusivity, with the unit of mm²/s. The dynamic mechanical properties of the sample were tested (DMA 242E, NETZSCH, Germany). The specific test mode was the three-point bending mode. The sample size was 40 mm × 8 mm × 2 mm. The test temperature range was from room temperature to 200 ℃. The heating rate was 5 ℃/min, the frequency was 1 Hz, and the atmosphere was air.

**Reference**s

[1]	T. Guo, D. Zhou, S. Deng, M. Jafarpour, J. Avaro, A. Neels, J. Heier, C. Zhang, *ACS Nano* **2023**, *17*, 3737.

[2]	D. Sheberla, J. C. Bachman, J. S. Elias, C. Sun, Y. Shao-Horn, M. Dincă, *Nature Materials* **2017**, *16*, 220.

[3]	Z. Zhang, Z. Cai, Z. Wang, Y. Peng, L. Xia, S. Ma, Z. Yin, Y. Huang, *Nano-Micro Letters* **2021**, *13*, 56.

[4]	H. Lv, Z. Yang, H. Pan, R. Wu, *Progress in Materials Science* **2022**, *127*, 100946.

[5]	Y. Hou, Z. Sheng, C. Fu, J. Kong, X. Zhang, *Nature Communications* **2022**, *13*, 1227.

[6]	Y. Li, X. Liu, X. Nie, W. Yang, Y. Wang, R. Yu, J. Shui, *Advanced Functional Materials* **2019**, *29*, 1807624.

[7]	C. Hu, Z. Mou, G. Lu, N. Chen, Z. Dong, M. Hu, L. Qu, *Physical Chemistry Chemical Physics* **2013**, *15*, 13038.

[8]	M. Dong, L. Zhou, J. Wang, G. Wang, X. Zhang, S. Guo, N. Wu, *Carbon* **2026**, *252*, 121414.

[9]	J. Tang, F. Guo, M. Ni, X. Guan, C. Liu, G. Ji, *Journal of Alloys and Compounds* **2025**, *1046*, 184891.

[10]	R. Shu, L. Nie, Z. Wan, *Journal of Alloys and Compounds* **2025**, *1010*, 177937.

[11]	X. Liang, Q. Xuan, H. Li, P. Ding, Y. Zhang, M. C. Koo, C. Liang, S. Yang, P. Zhao, D. Zhang, G. Wang, *Chemical Engineering Journal* **2025**, *523*, 168409.

[12]	L. Liang, Q. Li, X. Yan, Y. Feng, Y. Wang, H. Zhang, X. Zhou, C. Liu, C. Shen, X. Xie, *ACS Nano* **2021**, *15*, 6622.

[13]	W. Abdul, X. Zhao, W. Liu, Y. Sun, J. Guo, W. Wu, Z. Guo, J. Zhu, *Materials Today Communications* **2025**, *46*, 112942.

[14]	Y. Sui, N. Wu, Y. Liu, J. Lin, Y. Deng, S. Jiao, J. Liu, Z. Zeng, *Advanced Materials* **2026**, *36*, e14667.

[15]	Z. Wang, Z. Li, B. Li, A. Shi, L. Zhang, Y. Zhu, F. Ye, S. Yu, *Advanced materials (Deerfield Beach, Fla.)* **2024**, e2412605.

[16]	J. Qi, J. Zhang, Y. An, T. Zhang, R. Wang, W. Zhang, Y. Zhang, Y. Yang, L. Xia, *Carbon* **2025**, *232*, 119794.

[17]	S. Li, Y. Sun, F. Meng, X. Jiang, H. Yu, *Chemical Engineering Journal* **2024**, *498*, 155405.

[18]	H. Zhang, F. Li, N. Wu, L. Liu, X. Xie, H. Kimura, W. Du, Y. Liu, C. Hou, *Chemical Engineering Journal* **2025**, *523*, 168302.

[19]	Z. Tang, L. Xu, C. Xie, L. Guo, L. Zhang, S. Guo, J. Peng, *Nature Communications* **2023**, *14*, 5951.

[20]	R. Zhao, T. Gao, Y. Li, Z. Sun, Z. Zhang, L. Ji, C. Hu, X. Liu, Z. Zhang, X. Zhang, G. Qin, *Nature Communications* **2024**, *15*, 1497.

[21]	S. Cheng, D. Sheng, S. Mukherjee, W. Dong, Y. Huang, R. Cao, A. Xie, R. A. Fischer, W. Li, *Nature Communications* **2024**, *15*, 9077.

[22]	Y. Liu, J. Zhou, C. Li, H. Zhang, Y. Wang, Y. Yan, L. Duan, Z. Cheng, Y. Ma, Z. Yao, *Nature Communications* **2025**, *16*, 202.

[23]	J. Tao, Y. Yan, J. Zhou, J. Wang, P. Chen, R. Tan, L. Xu, H. Zhu, W. Zhu, H. Huang, X. Tao, Z. Yao, *Nature Communications* **2025**, *16*, 3163.

[24]	X. Zhang, X. Tian, N. Wu, S. Zhao, Y. Qin, F. Pan, S. Yue, X. Ma, J. Qiao, W. Xu, W. Liu, J. Liu, M. Zhao, K. K. Ostrikov, Z. Zeng, *Science advances* **2024**, *10*, eadl6498.

[25]	Z. Gao, A. Iqbal, T. Hassan, S. Hui, H. Wu, C. M. Koo, *Advanced Materials* **2024**, *36*, 2311411.

[26]	M. He, X. Zhong, X. Lu, J. Hu, K. Ruan, H. Guo, Y. Zhang, Y. Guo, J. Gu, *Advanced materials* **2024**, *36*, 2410186.

[27]	N. Qu, H. Sun, Y. Sun, M. He, R. Xing, J. Gu, J. Kong, *Nature Communications* **2024**, *15*, 5642.
